# Genome Assembly and Sex-Determining Region of Male and Female *Populus* × *sibirica*

**DOI:** 10.3389/fpls.2021.625416

**Published:** 2021-09-08

**Authors:** Nataliya V. Melnikova, Elena N. Pushkova, Ekaterina M. Dvorianinova, Artemy D. Beniaminov, Roman O. Novakovskiy, Liubov V. Povkhova, Nadezhda L. Bolsheva, Anastasiya V. Snezhkina, Anna V. Kudryavtseva, George S. Krasnov, Alexey A. Dmitriev

**Affiliations:** ^1^Engelhardt Institute of Molecular Biology, Russian Academy of Sciences, Moscow, Russia; ^2^Moscow Institute of Physics and Technology, Moscow, Russia

**Keywords:** *Populus*, poplar, sex determination, phylogeny, *de novo* genome assembly, Nanopore sequencing, pure high-molecular-weight DNA, *Populus*-specific repeat

## Abstract

The genus *Populus* is presented by dioecious species, and it became a promising object to study the genetics of sex in plants. In this work, genomes of male and female *Populus* × *sibirica* individuals were sequenced for the first time. To achieve high-quality genome assemblies, we used Oxford Nanopore Technologies and Illumina platforms. A protocol for the isolation of long and pure DNA from young poplar leaves was developed, which enabled us to obtain 31 Gb (N50 = 21 kb) for the male poplar and 23 Gb (N50 = 24 kb) for the female one using the MinION sequencer. Genome assembly was performed with different tools, and Canu provided the most complete and accurate assemblies with a length of 818 Mb (N50 = 1.5 Mb) for the male poplar and 816 Mb (N50 = 0.5 Mb) for the female one. After polishing with Racon and Medaka (Nanopore reads) and then with POLCA (Illumina reads), assembly completeness was 98.45% (87.48% duplicated) for the male and 98.20% (76.77% duplicated) for the female according to BUSCO (benchmarking universal single-copy orthologs). A high proportion of duplicated BUSCO and the increased genome size (about 300 Mb above the expected) pointed at the separation of haplotypes in a large part of male and female genomes of *P.* × *sibirica*. Due to this, we were able to identify two haplotypes of the sex-determining region (SDR) in both assemblies; and one of these four SDR haplotypes, in the male genome, contained partial repeats of the *ARR17* gene (Y haplotype), while the rest three did not (X haplotypes). The analysis of the male *P.* × *sibirica* SDR suggested that the Y haplotype originated from *P. nigra*, while the X haplotype is close to *P. trichocarpa* and *P. balsamifera* species. Moreover, we revealed a *Populus*-specific repeat that could be involved in translocation of the *ARR17* gene or its part to the SDR of *P*. × *sibirica* and other *Populus* species. The obtained results expand our knowledge on SDR features in the genus *Populus* and poplar phylogeny.

## Introduction

In angiosperm plants, only about 5% of species are dioecious, which implies that male and female flowers are developed on separate individuals (Yampolsky and Yampolsky, 1922; Renner, 2014). However, plants are characterized by very polymorphic mechanisms of sex determination, and diverse species are at different stages of sex chromosome evolution (Diggle et al., 2011; Charlesworth, 2016). A good example of polymorphism in a sex determination system is the genus *Populus*, which is presented by dioecious species. Most *Populus* species have an XY system of sex determination, where males are heterogametic, however, *Populus alba* has a ZW system with heterogametic females (Gaudet et al., 2008; Pakull et al., 2009, 2015; Paolucci et al., 2010; Kersten et al., 2014; Geraldes et al., 2015).

During the last years, extensive studies on sex determination in *Populus* species were performed: sex-specific DNA polymorphisms were identified and the sex-determining region (SDR) was mapped to the pericentromeric region of chromosome 19 in *Populus tremuloides*, *Populus tremula*, and *P. alba*; to the peritelomeric end of chromosome 19 in *Populus trichocarpa*, *Populus balsamifera*, and *Populus deltoides*; to the proximal end of chromosome 14 in *Populus euphratica* (Pakull et al., 2009, 2015; Paolucci et al., 2010; Kersten et al., 2014; Geraldes et al., 2015; McKown et al., 2017; Melnikova et al., 2019; Müller et al., 2020; Xue et al., 2020; Yang et al., 2020; Zhou et al., 2020b). Besides, it was shown that the SDR of *Populus* species with the XY system differs in size: the region consists of about 100 kb in *P. trichocarpa* (section *Tacamahaca*) and *P. deltoides* (section *Aigeiros*), while in *P. tremula* [section *Populus* (*Leuce*)] it is several times longer (Müller et al., 2020; Yang et al., 2020). However, it was revealed that the same gene, *ARABIDOPSIS RESPONSE REGULATOR 17* (*ARR17*) orthologue, is involved in sex determination in *Populus* species, including *P. alba*, *P. tremula*, *P. trichocarpa*, *P. davidiana*, *P. deltoides*, *P. euphratica* (Müller et al., 2020; Xue et al., 2020; Yang et al., 2020; Zhou et al., 2020b).

In the study of Muller et al., male-specific partial duplicates of *ARR17* were identified in the SDR of *P. tremula* and *P. trichocarpa*. It was suggested that two of the partial duplicates arranged as inverted repeats can form double-stranded RNA (dsRNA) and produce small RNAs that lead to silencing of *ARR17 via* DNA methylation. Using CRISPR-Cas9-mediated mutations in male and female aspen lines, it was shown that *ARR17* works as a sex switch – plants developed male flowers when *ARR17* was off and female ones when *ARR17* was on. In *P. alba*, which has the ZW system of sex determination, *ARR17* was deleted in males, while in the SDR of females three copies of *ARR17* were present (Müller et al., 2020).

In the work of Xue et al., two male-specific genes were revealed in the SDR of *P. deltoides*. One of these genes (named *FERR-R*, referred to as *ARR17* partial repeats in most of the other studies) was suggested to repress the formation of female generative organs through methylation of the *FERR* gene (referred to as *ARR17* in most other studies) and cleavage of its transcript. Another male-specific gene (named *MSL*), as was stated, transcribed a long non-coding RNA (lncRNA) that promoted male functions. The role of *FERR* and *MSL* in the development of reproductive organs was confirmed in transformed *Arabidopsis*: *FERR* promoted the development of the female reproductive organs, while *MSL –* the male ones. Thus, two male-specific genes (*FERR-R* and *MSL*) could be involved in sex determination of *P. deltoides*. However, *MSL* is not necessary for androecia development in all *Populus* species, as the complete sequence of this gene was identified in males of *P. deltoides* (section *Aigeiros*) and *P. simonii* (section *Tacamahaca*) but not *P. davidiana* and *P. tremula* [section *Populus* (*Leuce*)] (Xue et al., 2020).

A detailed analysis of the SDR of *P. trichocarpa* was performed by Zhou et al. It was shown that the SDR size is about 120 kb, 50 kb of which are male-specific (absent in females) and primarily include fragments of Gypsy LTR-retrotransposons. Five partial repeats (*ARM-1*, *ARM-2*, *ARM-3*, *ARM-4a*, and *ARM-4b*) of *ARR17* (referred to as *PtRR9* or Po14v11g057342m) in the SDR had more than 90% of sequence identity but varied in parts of *ARR17* that they included. Expression from these partial repeats of *ARR17* was detected in floral tissues of male plants at different stages of development. Besides, genes encoding T-complex protein 1 subunit gamma (TCP), Chloride channel protein CLC-c (CLC), DNA-methyltransferase 1 (MET1), and Leucine-rich repeat-containing protein (LRR) were located in an SDR part that was present in both Y and X variants (Zhou et al., 2020b).

In recent studies on sex determination in *Populus* species, third-generation sequencing platforms [Oxford Nanopore Technologies (ONT) and Pacific Biosciences (PacBio)] with a read length of up to hundreds of thousands of nucleotides were used that enabled researchers to gain breakthrough knowledge about the SDR (Müller et al., 2020; Xue et al., 2020; Yang et al., 2020; Zhou et al., 2020b). The analysis of SDR polymorphism in different *Populus* species will give us new information on general and specific features of this genomic region. Therefore, further studies concerning male and female distinctions in poplars and aspens are necessary, and genomic data for different species of the genus *Populus* obtained using long reads will be more informative for the identification of species-specific sequences. Poplars are wind-pollinated trees, and different species are easily crossed, which leads to the emergence of natural interspecific hybrids and a high level of genetic diversity (Rae et al., 2007; Roe et al., 2014; Jiang et al., 2016). In cities of European Russia, poplar hybrids are more common in comparison with native species. *Populus* × *sibirica*, which is an intersectional hybrid, likely between *P. nigra* (section *Aigeiros*) and a poplar from section *Tacamahaca* (probably hybrid of *P. laurifolia* and *P. suaveolens*), is one of the most common ones (Mayorov et al., 2012; Kostina and Nasimovich, 2014; Kostina et al., 2017). Poplars, particularly *P.* × *sibirica*, are actively used in landscaping of Russian cities due to the tolerance to unfavorable atmospheric and soil conditions and are particularly important for the improvement of ecology by planting these trees near the streets with high transport traffic (Kostina et al., 2017). However, problems associated with fluff, which is produced only by female trees, limit the use of poplars in cities. Therefore, studies of sex-associated differences in *P.* × *sibirica* have not only basic significance but the applied importance. The present study aimed to obtain the first genome assemblies of male and female individuals of *P.* × *sibirica*, identify SDR sequences, and compare them with other poplar species.

## Materials and Methods

### Plant Material

*Populus* × *sibirica* trees used in the present study grow in Moscow, Russia within the region from 55°42′14″N to 55°42′23″N and from 37°35′05″E to 37°34′50″E. Young leaves were collected from male and female plants of *P.* × *sibirica* during the beginning of flowering and immediately frozen in liquid nitrogen. Plant material was stored at −70°C until DNA isolation.

### DNA Extraction and Purification

Young *P.* × *sibirica* leaves with a mass of 0.2 g were triturated in a mortar with liquid nitrogen. Then, we added 4 ml of the Carlson lysis buffer [100 mM Tris–HCl pH 9.5 (VWR Life Science, United States); 2% CTAB (VWR Life Science); 1.4 M NaCl (Scharlab, Spain); 1% PEG 8000 (PanReac AppliChem, Germany); 20 mM EDTA (Promega, United States)] that was prewarmed to 65°C and supplemented with 12 μl of β-mercaptoethanol (BioRad, United States) and 0.04 g of PVP K30 (PanReac AppliChem). The homogenate was incubated in a “Gnom” thermostat (DNA Technology, Russia) at 65°C for 1 h, with stirring every 10 min. Next, an equal volume of chloroform (Acros Organics, United States) was added to the homogenate followed by stirring the mixture on a Thermolyne Maxi Mix III Type 65800 shaker (Thermo Fisher Scientific, United States) at 800 rpm for 10 min and its centrifugation on a 5418R microcentrifuge (Eppendorf, Germany) using the following parameters: 10000 *g*, 10 min, 4°C. The aqueous phase was transferred to a clean tube with the addition of 0.2 volume of 5× CTAB buffer (5% CTAB, 350 mM EDTA) and incubated at 65°C for 10 min. After that, an equal volume of chloroform was introduced, and the mixture was stirred on a shaker for 10 min and centrifuged with the following parameters: 10000 *g*, 10 min, 4°C. The aqueous phase was transferred to a clean tube with the addition of 0.1 volume of 5× CTAB buffer and incubated at 65°C for 10 min. After that, an equal volume of chloroform was introduced, and the mixture was stirred on a shaker for 10 min and centrifuged with the following parameters: 10000 *g*, 10 min, 4°C. The aqueous phase was transferred to clean tubes with the addition of 2 volumes of the buffer for DNA precipitation (1% CTAB, 50 Mm Tris–HCl pH 8.0, 10 Mm EDTA) and incubated at room temperature for 1 h. It was then centrifuged at 10000 *g* for 15 min at room temperature. Next, the supernatant was collected gently without disturbing the precipitate. The DNA pellet was air-dried for 5 min, dissolved in 2 ml of prewarmed to 60°C G-buffer from the Blood and Cell Culture DNA Mini Kit (Qiagen, United States), and incubated at 60°C for 10 min.

To the DNA sample in the G-buffer, 4 μl of RNase A (100 mg/ml; 7000 units/ml; Qiagen) were added and incubated at 37°C for 30 min. To this, 25 μl of proteinase K (> 600 mAU/ml; Qiagen) were introduced and incubated at 50°C for 40 min. Further, DNA was purified according to the Blood and Cell Culture DNA Mini Kit (Qiagen) protocol. To the DNA elution, 0.7 volume of isopropanol was added, and the mixture was stirred until DNA strands appeared. The strands were neatly wrapped around a glass rod, transferred to a tube containing DNA dilution buffer (Evrogen, Russia), and incubated at 50°C for 60 min. The DNA quality and concentration were assessed on a NanoDrop 2000C spectrophotometer (Thermo Fisher Scientific). The DNA concentration was also evaluated on a Qubit 2.0 fluorometer (Life Technologies, United States). The proximity of concentration values measured with Qubit and Nanodrop served as an additional criterion of DNA purity. The assessment of DNA length and control of the absence of RNA were performed by electrophoresis in a 0.8% agarose gel (Lonza, Switzerland).

### DNA Library Preparation and Sequencing on the ONT Platform

To remove short DNA fragments (up to 10 kb), the Short Read Eliminator Kit (Circulomics, United States) was used. Then, the DNA sample was diluted to a concentration of 60 ng/ml in a final volume of 50 μl of DNA dilution buffer (Evrogen) and further purified with AMPure XP beads (Beckman Coulter, United States) in a ratio of 1:0.7 (sample:beads).

Preparation of the libraries was performed using the SQK-LSK109 Ligation Sequencing Kit (ONT, United Kingdom) for 1D genomic DNA sequencing. Minor modifications were introduced to the recommended protocol for library preparation by increasing the incubation time to 20 min at 20°C at the step of combined recovery of DNA using the NEBNext Ultra II End Repair/dA-Tailing Module (New England Biolabs, United Kingdom) and NEBNext FFPE DNA Repair Mix (New England Biolabs). Besides, the incubation time at the step of ligation was increased to 60 min. Sequencing was performed on MinION (ONT) with FLO-MIN-106 R9.4.1 and FLO-MIN-110 R10.0 flow cells (ONT).

### DNA Library Preparation and Sequencing on the Illumina Platform

DNA fragmentation was performed on an S220 ultrasonic homogenizer (Covaris, United States), and the NEBNext Ultra II DNA Library Prep Kit for Illumina (New England Biolabs) was used for DNA library preparation according to the manufacturer’s protocol. The quality and concentration of libraries were evaluated on 2100 Bioanalyzer (Agilent Technologies, United States) and Qubit 2.0 (Life Technologies), respectively. Sequencing was performed on HiSeq 2500 (Illumina, United States) with a read length of 125+125 bases.

### Genome Assembly

The obtained MinION fast5 files were processed using Guppy 3.6.1 with high-accuracy flip-flop algorithms (dna_r9.4.1_450bps_hac.cfg and dna_r10_450bps_hac.cfg configuration files for R9.4.1 and R10.0 flow cell data, respectively). Then, adapter sequences were removed using Porechop^1^, and low-quality reads (average *Q* < 6) were filtered out with Trimmomatic 0.32 (Bolger et al., 2014).

Genome assemblies based on the Nanopore reads were performed with five tools: Canu 2.1 (Koren et al., 2017), Flye 2.8 (Kolmogorov et al., 2019), Raven 1.1.10 (Vaser and Šikić, 2020), Shasta 0.5 (Shafin et al., 2020), and wtdbg2 2.5 (Ruan and Li, 2020). The default parameters were used, except for the minimal read length for Shasta (was set to 3000 bp) and expected genome size for Flye and wtdbg2 (was set to 500 Mb). The statistics for the genome assemblies were calculated using QUAST 5.0.2 (Gurevich et al., 2013). Misassembly rates between our assemblies and the *P. trichocarpa* “Stettler 14” genome v1.1^2^ (Hofmeister et al., 2020) were also evaluated using QUAST.

To improve the accuracy of contigs, the assemblies were polished with Racon 1.4.3 (two iterations) (Vaser et al., 2017) and Medaka 1.0.3^3^ using Nanopore reads. Since Medaka is specific to the type of the used flow cell, the data derived only from the R9.4.1 flow cells were used for polishing with this tool. Assembly polishing using Illumina reads was performed with the POLCA tool from MaSuRCA 3.4.1 assembler (Zimin et al., 2017; Zimin and Salzberg, 2020). Assembly completeness was evaluated with BUSCO v4, the embryophyta_odb10 dataset (Seppey et al., 2019).

### Profiles of Self-Coverage of Genome Assemblies

The profiles of self-coverage were obtained by aligning genomic sequences to themselves with blastn 2.11.0 (default search parameters) (Zhang et al., 2000). Then, the alignment results were parsed and the percentage of bases covered 1, 2, 3, 4, or 5+ times was calculated. We considered only alignments with the length > 300 bp, identity > 75%, and bitscore > 1000. The divergence between alleles (heterozygosity) was also calculated based on blastn results.

### Analysis of the SDR and *ARR17* Gene

First, we analyzed our assemblies of male and female *P.* × *sibirica* with QUAST using the male *P. trichocarpa* “Stettler 14” genome as a reference [the assembled contigs were mapped to the “Stettler 14” genome with minimap2 v. 2.17, (Li, 2018)] and looked for contigs that were mapped to the SDR (Chr18:16,200,000-16,320,000) and *ARR17* gene (PtStettler14.19G117100, Chr19:15,907,431-15,910,397) of “Stettler 14”. It should be noted that in the “Stettler 14” genome assembly, the SDR was mapped to chromosome 18, but the correct place of the SDR is on chromosome 19 according to the genetic map (Zhou et al., 2020b); however, the sequence itself provides an accurate representation of the SDR and can be used for the analysis. Second, the SDR and *ARR17* of “Stettler 14” were mapped to our assemblies with blastn. Both methods gave similar results and indicated contigs that contained the SDR and *ARR17* gene in *P.* × *sibirica* genome assemblies. For the identification of *MSL*, the *MSL* sequence of male *P. deltoides* [GCA_014884945.1, (Xue et al., 2020)] was mapped to our assemblies with blastn.

The LAST v.1066 aligner (Kielbasa et al., 2011) was used to compare SDR haplotypes of male and female *P.* × *sibirica* with the SDR of male *P. trichocarpa* “Stettler 14” visually. Multiple alignment of the *ARR17* gene and *ARR17* inverted repeats was done with MUSCLE (max. 50 iterations) in MEGA-X (Kumar et al., 2018) based on our assemblies of male and female *P.* × *sibirica* genomes and data from Supplementary File 2 from the article by Müller et al. (2020). In our male *P.* × *sibirica* genome assembly, *ARR17* inverted repeats were identified by a fragment of the *HEMA* gene between them (Müller et al., 2020). Maximum-likelihood phylogenetic tree reconstruction was also performed in MEGA-X with default parameters (Tamura-Nei substitution model, uniform rates). Multiple alignment and phylogenetic analysis of *MET1* (PtStettler14.18G127700), *CLC* (PtStettler14.18G127800), or *TCP* (PtStettler14.18G127900) genes from our assemblies were performed in the same way (except that ClustalW was used instead of MUSCLE). For the phylogenetic analysis of the *ARR17* gene and five *ARR17* partial repeats of *P.* × *sibirica* and its close poplar species with available high-quality genomes, sequences were extracted by mapping the *ARR17* gene with surrounding regions (1 kb in both directions) from the “Stettler 14” genome against our assembly of male *P.* × *sibirica* and assemblies of males of *P. trichocarpa* (Phytozome: “Stettler 14”), *P. deltoides* (NCBI: GCA_014884945.1), and *P. simonii* (NCBI: GCA_007827005.2). Then sequences were trimmed so that they included only common regions that are present in all analyzed *ARR17* genes (including an upstream region) and *ARR17* partial repeats (the resulting sequences are presented in Supplementary Data 1). Multiple alignment and phylogenetic analysis were performed as described above.

For the comparison of the SDR and *ARR17* gene between *P.* × *sibirica* and species from sections *Aigeiros* and *Tacamahaca*, we used Illumina whole-genome sequencing (WGS) data obtained by us for *P.* × *sibirica* and by other researchers for *P. nigra*, *P. deltoides* (section *Aigeiros*) and *P. trichocarpa*, *P. balsamifera*, *P. simonii*, *P. cathayana*, *P. koreana* (section *Tacamahaca*) (NCBI SRA, in total, 70 runs from six BioProjects: PRJNA628142, PRJNA646700, PRJNA276056, PRJNA241273, PRJNA303130, and PRJNA540895). SRA data were prefetched and then unpacked with fastq-dump 2.10.9 (NCBI SRA toolkit). Reads were trimmed and adapter sequences were removed with Trimmomatic. Next, the reads were mapped to the *P. trichocarpa* “Stettler 14” genome or only its SDR as a reference with BWA 0.7.17. The derived BAM files were sorted with Samtools 1.10 and reordered with ReorderSam from Picard tools 2.21.3. Then, read groups were added (AddOrReplaceReadGroups from Picard tools), and mate CIGAR fields were placed in BAM files (FixMateInformation from Picard tools). Finally, duplicated reads were marked with the MarkDuplicatesWithMateCigar utility from Picard tools. Single nucleotide polymorphism (SNP) calling was performed using FreeBayes 1.3.2 for the joint pool of processed BAM files with the following parameters: minimal mapping quality – 10, minimal base-calling quality – 12, minimal base-calling quality sum – 20, minimal coverage – 5, minimal variant allele fraction – 0.1, ploidy – 4. These lowered thresholds were selected because of the presence of low-coverage and low-quality GaIIx sequencing data in the dataset. The search for polymorphisms was limited to regions Chr19:15,907,431-15,910,397 (*ARR17*), Chr18:16,290,253-16,308,089 (the locus of *ARR17* partial repeats), and Chr18:16,200,000-16,320,000 (SDR) of *P. trichocarpa* “Stettler 14” genome. We extracted variant allele fraction (VAF) profiles from the derived VCF file and calculated cross-sample Euclidean distances (the positions that were covered with less than 5 reads for a current sample were excluded). Clustering was performed using Ward’s minimum variance method with squared distances (‘ward.D2’ method in R 3.6.2). Dendrogram and MDS (multidimensional scaling) plot visualization was performed using ggtree and ggplot2 packages for R. The similar analysis (the same samples and the same tools) was also performed with the male *P.* × *sibirica* genome assembly as a reference [polymorphism search was limited to regions from 1,090,323 to the end of contig (1,200,738) for tig00001299 (Y SDR haplotype) and from 1,585,216 to the end of contig (1,676,203) for tig00000650 (X SDR haplotype)].

The search for ORFs was performed using TransDecoder.LongOrfs 5.5.0 (Haas et al., 2013). The functional analysis of predicted proteins was carried out with InterPro 85.0 web service (Blum et al., 2021).

## Results

### Genome Assembly of Male and Female *P.* × *sibirica*

In this study, we performed genome sequencing of male and female individuals of *P.* × *sibirica* on the ONT platform, as the use of long reads generated on third-generation sequencers is highly desirable for obtaining high-quality genome assemblies (Kyriakidou et al., 2018; van Dijk et al., 2018). The quality of DNA is crucial for Nanopore sequencing (Lu et al., 2016), therefore, we developed a protocol for the isolation of long high-purity DNA from poplar leaves and obtained the DNA of approximately 50 kb with A260/A280 of 1.7-2.0 and A260/A230 of 2.0-2.2. The concentrations measured with a Nanodrop spectrophotometer (Thermo Fisher Scientific) and a Qubit fluorometer (Life Technologies) had similar values, which was an important criterion of high purity.

Genome sequencing on the ONT platform brought about 31 Gb (after adapter trimming and quality filtering) for the male poplar (three runs with R9.4.1 and one run with R10.0) and 23 Gb for the female one (two runs with R9.4.1 and one run with R10.0) with N50 of about 21 and 24 kb for the male and female, respectively. The size of the *P. trichocarpa* genome is about 500 Mb (Tuskan et al., 2006), thus, approximately 62× and 46× genome coverage was obtained for the male and female, respectively.

It is known that different assemblers provide different results depending on sequencing data volume and quality, as well as genome size and complexity (Jung et al., 2019; Krasnov et al., 2020; Murigneux et al., 2020; Vaser and Šikić, 2020). Therefore, genome assembly of *P.* × *sibirica* individuals was performed using five tools (Canu, Flye, Raven, Shasta, and wtdbg2) for further comparison of the results (Table 1). The lengths of the obtained assemblies for male and female plants were close – about 500-550 Mb for Raven and wtdbg2, about 700 Mb for Flye and Shasta, and about 800 Mb for Canu. Canu gave the greatest length of the largest contig (7.7 Mb and 3.6 Mb for the male and female genome assemblies, respectively), while the smallest length was given by Shasta (1.4 Mb for both). Canu was also the best in terms of Nx and Lx statistics and produced the highest N50 value of 1.5 Mb for the male and 0.5 Mb for the female (Table 1).

**TABLE 1:** QUAST statistics for the genome assemblies of male and female *P.* × *sibirica* plants.

Feature	Canu 2.1	Flye 2.8	Raven 1.1.10	Shasta 0.5	wtdbg2 2.5
	**Male**				

Total assembly length, Mb	818	704	513	704	569
Number of contigs	1,720	18,084	2,306	12,411	9,374
Largest contig, Mb	7.7	2.4	3.4	1.4	3.3
GC, %	33.91	34.06	33.11	33.46	34.33
N50, kb	1,494	200	304	146	150
L50	154	848	454	1,267	705

	**Female**				

Total assembly length, Mb	816	691	495	701	487
Number of contigs	3,354	13,961	2,198	10,370	8,182
Largest contig, Mb	3.6	2.5	2.7	1.4	2.6
GC, %	33.65	33.83	33.20	33.36	33.35
N50, kb	509	210	325	201	159
L50	417	722	404	981	585

**FIGURE 1:**
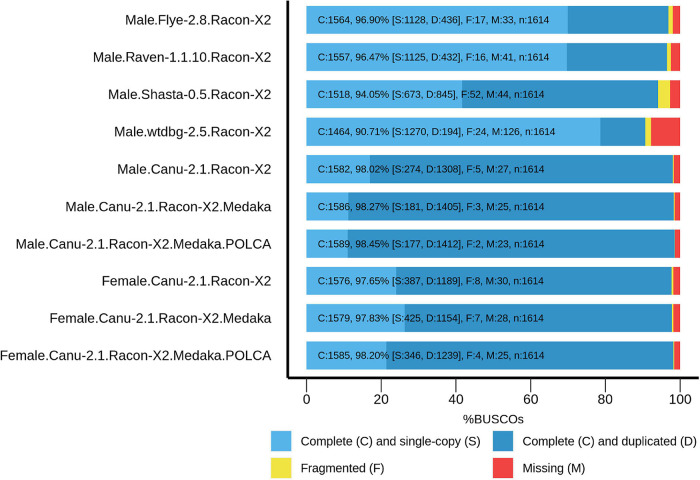
BUSCO assessment results for the genome assemblies of male and female *P.* × *sibirica* plants. Results for Canu, Flye, Raven, Shasta, and wtdbg2 assemblers and Racon, Medaka, and POLCA polishers are presented.

To improve contig accuracy, the assemblies were polished twice with Racon using Nanopore reads. For further comparison, we assessed misassembly rates between our polished male genome assemblies and the genome of male *P. trichocarpa* “Stettler 14” using QUAST (Table 2). Since two different *Populus* species were compared, a significant number of misassemblies was expected. Having performed such an analysis, we could mainly compare *P.* × *sibirica* assemblies performed with different tools, that is, find out which of the assemblers gave the best result in terms of consistency of a corresponding assembly with the *P. trichocarpa* “Stettler 14” genome and its relative completeness. In most key parameters (covered reference genome fraction, completed reference genomic features (i.e., genes, transcripts, exons), LG and NG statistics), Canu gave the best results (Table 2). We also evaluated the completeness of our assemblies using BUSCO, which assesses the presence of benchmarking universal single-copy orthologs (BUSCO), and Canu provided the highest results again (Figure 1). Thus, the assemblies of male and female *P.* × *sibirica* genomes performed with Canu were considered the most complete and accurate and used for further analysis.

**TABLE 2:** Consistency between the obtained genome assemblies of male *P.* × *sibirica* and the genome of male *P. trichocarpa* “Stettler 14”.

Feature	Canu 2.1	Flye 2.8	Raven 1.1.10	Shasta 0.5	wtdbg2 2.5
Covered reference genome fraction, %	47.2	42.8	40.2	45.7	35.7
Duplication ratio	1.8	1.3	1.3	1.5	1.2
Misassemblies per 1 Mb	2.3	1.6	2.1	1.4	1.7
Misassemblies	1,893	1,116	1,088	984	952
Reference genomic “features” complete	727,803	678,370	665,573	702,592	564,900
Largest alignment, kb	953	676	763	171	509
Total aligned length, Mb	327	222	198	264	167
NG50, kb	2,963	402	427	254	338
NG75, kb	2,145	262	274	176	140
LG50	49	290	273	468	291
LG75	88	597	565	926	764

*The data are presented for the *P.* × *sibirica* genome assemblies polished with Racon (two iterations). NG50/NG75 is the maximum length for which the subset of contigs of that length or longer covers at least 50%/75% of the reference genome (*P. trichocarpa* “Stettler 14”). LG50/LG75 is the number of contigs with a length equal to or greater than NG50/NG75, i.e., the minimal number of contigs that cover 50%/75% of the reference genome.*

High percentages of duplicated BUSCO (81% for the male genome assembly and 74% for the female one, Figure 1) and increased genome sizes (about 300 Mb above the expected 500 Mb for both) indicated the separation of haplotypes for most of male and female *P.* × *sibirica* genomes. To test this assumption, we performed self-alignment of our *P.* × *sibirica* assemblies and several genomes of other *Populus* species from the NCBI database: GCA_007827005.2 (*P. simonii*) (Murigneux et al., 2020), GCA_014884945.1 (*P. deltoide*s) (Xue et al., 2020), GCA_014885025.1 (*P. deltoide*s) (Xue et al., 2020), GCA_014885075.1 (*P. davidiana)* (Xue et al., 2020), GCA_015708895.1 (*P. ilicifolia*) (Chen et al., 2020), GCA_015852605.1 (*P. deltoides*), GCA_000495115.1 (*P. euphratica*) (The results are presented in Supplementary Data 2). In our male and female *P.* × *sibirica* assemblies, about 45% of sequences aligned against themselves two times, and this percentage was significantly higher than the values for the other 7 genomes, which were about 15-20%. At the same time, only about 15% of a genome aligned against itself one time for both *P.* × *sibirica* assemblies, while these values were about 55-70% for the other analyzed *Populus* species (Supplementary Data 2). For *P.* × *sibirica*, the number of mismatches between alleles (or allele pairs) was about 130 per 1 kb, the number of gaps — about 31 per 1 kb, the overall identity – about 83%. Considering that the assemblies of male and female *P.* × *sibirica* had increased size and a high proportion of duplicated BUSCO and mainly comprised sequences that aligned against themselves two times, it can be concluded that a large part of both assemblies was presented by two haplotypes.

The obtained male and female *P.* × *sibirica* assemblies were additionally polished using Nanopore reads with Medaka, and their completeness increased from 98.02 to 98.27% for the male and from 97.65 to 97.83% for the female. Further polishing of the assemblies using Illumina reads (36 million paired-end 2×125-bp reads were obtained for the male *P.* × *sibirica* and 52 million reads – for the female one) was performed with POLCA that increased the BUSCO completeness up to 98.45% (87.48% duplicated) for the male and 98.20% (76.77% duplicated) for the female (Figure 1). According to BWA mapping statistics, less than 0.2% of Illumina reads were mapped to more than one genomic region, so POLCA polishing did not lead to haplotype unification, which was important for further analysis.

### Analysis of the SDR of *P.* × *sibirica*

**FIGURE 2:**
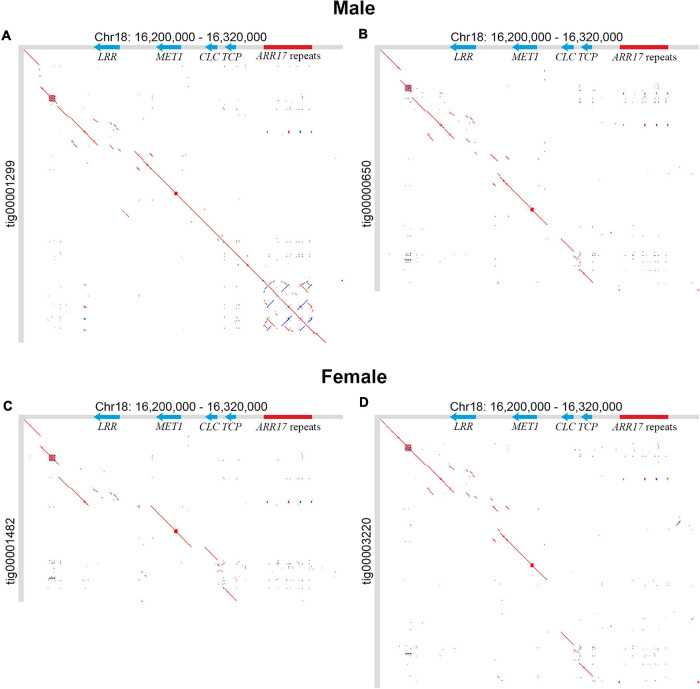
Comparison of SDR variants of male and female *P.* × *sibirica* plants with the SDR of male *P. trichocarpa* “Stettler 14.” **(A)** The Y SDR haplotype of the male, where partial repeats of *ARR17* are located in the bottom right corner. **(B)** The X SDR haplotype of the male. **(C,D)** Variants of the X SDR haplotype of the female. Tig00001482 is presented in reverse complement orientation. *LRR*, *MET1*, *CLC*, and *TCP* genes and *ARR17* partial repeats are marked on the SDR of “Stettler 14”.

Next, the search for the SDR in our male and female *P.* × *sibirica* assemblies was performed. We revealed two contigs (tig00001299 and tig00000650) in the male genome assembly and two contigs (tig00001482 and tig00003220) in the female one that contained sequences similar to the poplar SDR characterized by other researchers (Müller et al., 2020; Xue et al., 2020; Yang et al., 2020; Zhou et al., 2020b). The sequences of four SDR-containing contigs are presented in Supplementary Data 3. These sequences were aligned against the SDR of male *P. trichocarpa* “Stettler 14” (Chr18:16,200,000-16,320,000) and the following nucleotide positions corresponded to the SDR: from 1,090,323 to the end of contig (1,200,738) for tig00001299; from 1,585,216 to the end of contig (1,676,203) for tig00000650; from the start of contig to 68,843 for tig00001482 (reverse complement orientation); from 470,590 to the end of contig (571,850) for tig00003220. The comparison of four *P.* × *sibirica* SDR variants with the SDR of *P. trichocarpa* “Stettler 14” was performed using the alignments received with LAST (Figure 2). The sequence within the male tig00001299 comprised partial repeats of the *ARR17* gene (Figure 2A) and, therefore, was the Y haplotype of the *P.* × *sibirica* SDR. The sequence within the male tig00000650 had no partial repeats of *ARR17* (Figure 2B); so, this contig carried the X SDR haplotype of the male *P.* × *sibirica*. In the female genome assembly, both tig00001482 and tig00003220 had no partial repeats of *ARR17* (Figures 2C,D) and, hence, contained X SDR haplotypes. Thus, since we did not purge haplotypes, we were able to identify two SDR variants in both male and female genome assemblies. For all the three X SDR haplotypes of *P.* × *sibirica*, we revealed significant variation in intergenic regions (between the *TCP*, *CLC*, and *MET1* genes) and in the *LRR* gene region (Figure 2). These regions contained long sequences that differed from each other and those of “Stettler 14.” As can be seen from Figure 2, tig00000650 (male X) and tig00003220 (female X) were more similar to each other than to tig00001482 (female X). At the same time, the Y SDR variants of *P.* × *sibirica* and *P. trichocarpa* were much closer to each other than to the X SDR haplotypes of *P.* × *sibirica* that could be associated with suppression of recombination between Y and X variants of the SDR. We performed clustering of the Y and X SDR haplotypes of *P.* × *sibirica* based on the *TCP*, *CLC*, and *MET1* gene sequences (Supplementary Data 4). For all the three genes, tig00000650 (male X) clustered with tig00003220 (female X), and tig00001482 (female X) was also closer to these two X variants than to tig00001299 (male Y). The similarity of tig00000650 (male X) and tig00003220 (female X) could be associated with the same parental species of *P.* × *sibirica*, which probably made a substantial contribution to the differences between the SDR haplotypes of the same plant. It is worth noting that tig00001482 (female X) had some similarities to tig00001299 (male Y) – several common to them but different from tig00000650 (male X) and tig00003220 (female X) SNPs and short indels were identified. This is also probably associated with common parental species.

We did not find the complete *MSL* gene sequence in the SDR of the male *P.* × *sibirica –* only short regions with partial homology to *MSL* of *P. deltoides* were observed. The complete sequence of this gene was earlier identified in males of *P. deltoides* (section *Aigeiros*) and *P. simonii* (section *Tacamahaca*) but not *P. davidiana* and *P. tremula* [section *Populus* (*Leuce*)] (Xue et al., 2020). In the male *P. trichocarpa* “Stettler 14” genome, we also revealed a region (Chr18:16,316,742-16,319,611) with high similarity to *MSL*. The Y SDR haplotype of the male *P.* × *sibirica* probably originated from *P. nigra* (section *Aigeiros*) and, in this poplar species, the complete *MSL* may be absent; however, further studies are needed to solve this issue, first of all, by obtaining a high-quality genome assembly of *P. nigra* and searching for *MSL* in it.

### Phylogenetic Analysis of *P.* × *sibirica*

**FIGURE 3:**
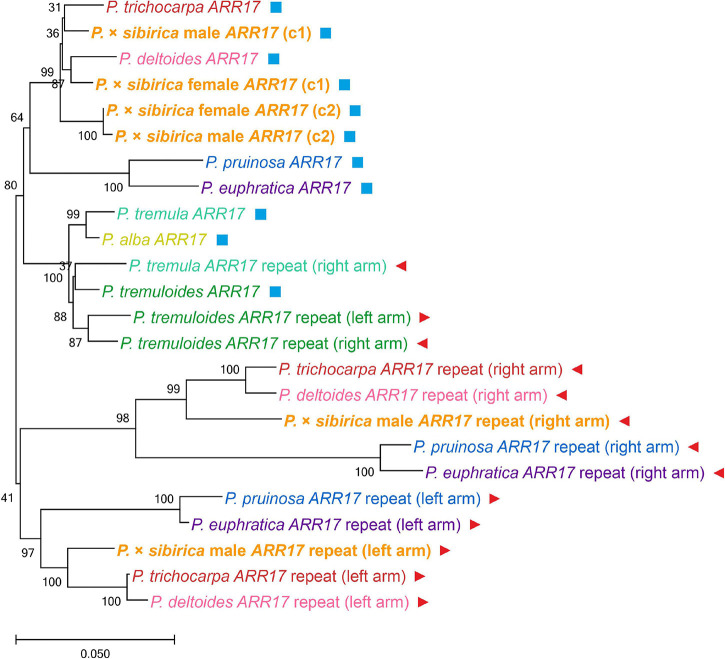
Clusterization of *Populus* species based on nucleotide sequences of the *ARR17* gene and *ARR17* inverted repeats. *ARR17* genes designated *ARR17*; in *P.* × *sibirica* genome assemblies, two allelic variants of the *ARR17* gene were identified [designated *ARR17* (c1) and *ARR17* (c2)]. *ARR17* inverted repeats designated *ARR17* repeat (left arm) and *ARR17* repeat (right arm). *P. tremula ARR17* repeat (left arm) is not presented as this sequence was truncated and excluded from the analysis. As *P. alba* has a ZW system of sex determination, where the *ARR17* gene is deleted in males and *ARR17* inverted repeats were not identified, data are presented only for the female *ARR17* gene. Additionally, *ARR17* genes are marked with blue squares and *ARR17* inverted repeats – with red triangles.

To determine the relationship between the key *Populus* sex regulators, *ARR17* and *ARR17* inverted repeats, from our *P.* × *sibirica* assemblies and the genomes of other *Populus* species, we performed a phylogenetic analysis (sequences corresponding to 7 *Populus* species (*P. trichocarpa*, *P. deltoides*, *P. pruinosa*, *P. euphratica*, *P. tremula*, *P. tremuloides*, and *P. alba*) were taken from Supplementary File 2 from the article by Müller et al., 2020; Figure 3). In our male and female *P.* × *sibirica* assemblies, two allelic variants of the *ARR17* gene were identified [designated *ARR17* (c1) and *ARR17* (c2)] – in tig00000357, tig00001424 for the male and tig00001689, tig00003114 for the female. These sequences formed a cluster with *ARR17* of *P. trichocarpa* and *P. deltoides*, and *ARR17* (c1) of male and female *P.* × *sibirica* were in one subcluster with *ARR17* of *P. trichocarpa* and *P. deltoides*, while *ARR17* (c2) were in another subcluster. The left arm of *ARR17* inverted repeats of the male *P.* × *sibirica* [designated *ARR17* repeat (left arm)] clustered with the left arms of *P. trichocarpa* and *P. deltoides*, and the right arm of *ARR17* inverted repeats [designated *ARR17* repeat (right arm)] – with the right arms of *P. trichocarpa* and *P. deltoides*; however, *P. trichocarpa* and *P. deltoides* were closer to each other than to *P.* × *sibirica* in both the “*ARR17* repeat (left arm)” group and the “*ARR17* repeat (right arm)” group (Figure 3). This analysis showed that *P.* × *sibirica* is phylogenetically closer to *P. trichocarpa* and *P. deltoides* than to the other 5 analyzed *Populus* species (*P. euphratica*, *P. pruinosa*, *P. alba*, *P. tremula*, and *P. tremuloides*) and also that *P. trichocarpa* and *P. deltoides* are closer to each other than to *P.* × *sibirica*. We also performed phylogenetic analysis for the *ARR17* gene and five *ARR17* partial repeats of males of *P.* × *sibirica*, *P. trichocarpa*, *P. deltoides*, and *P. simonii*, which have the same sex-determination system and for which high-quality genome assemblies are available. The results are presented in Supplementary Data 5. Six clusters were identified: a cluster of *ARR17* genes and five clusters of *ARR17* partial repeats grouped by their numbers – 1, 2, 3 (left arm), 4 (right arm), and 5 (the first repeat is the closest to the end of the chromosome and the subsequent ones are located further from the end in ascending order, according to the “Stettler 14” genome assembly). Thus, clusters were formed in accordance with genome regions but not with the classification of poplar individuals into different species, which indicates that divergence of the studied species occurred after the formation of sex chromosomes. Based on *ARR17* and its five partial repeats, *P. trichocarpa*, *P. deltoides*, and *P. simonii* were closer to each other than to *P.* × *sibirica*, except for *ARR17* (c1) that was located within the *P. trichocarpa*, *P. deltoides*, and *P. simonii* group.

**FIGURE 4:**
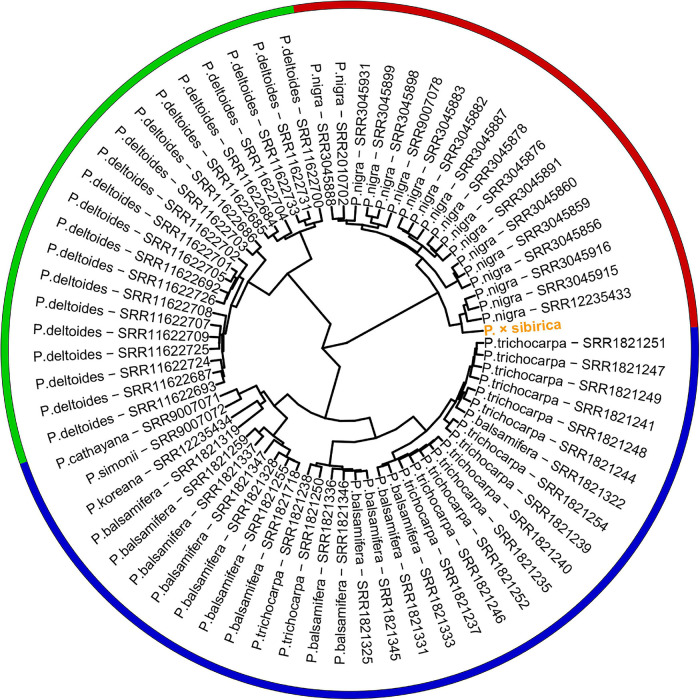
Clusterization of 70 *Populus* males and the male *P.* × *sibirica* based on Illumina WGS data aligned against the male *P. trichocarpa* “Stettler 14” genome with a further search for polymorphisms in the SDR. Three main clusters are marked with red (*P. nigra* and *P.* × *sibirica*), green (*P. deltoides*), and blue (*Tacamahaca* species).

Genome assemblies are available for a limited number of *Populus* species, and we did not find assembled genomes of *P. nigra*, which is a probable ancestor of *P.* × *sibirica*, and two other species that presumably participated in the formation of *P.* × *sibirica* (*P. laurifolia* and *P. suaveolens*). Therefore, we took Illumina WGS data to evaluate the relationship of the male *P.* × *sibirica* with 70 males from sections *Aigeiros* (*P. nigra* and *P. deltoides*) and *Tacamahaca* (*P. trichocarpa*, *P. balsamifera*, *P. simonii*, *P. cathayana*, *P. koreana*; unfortunately, we did not find WGS data for *P. laurifolia* and *P. suaveolens*) and aligned these data against the *P. trichocarpa* “Stettler 14” genome as a reference. When the *ARR17* gene (Chr19:15,907,431-15,910,397) was used for clustering, we observed that the samples formed 3 groups: first – *P. deltoides*, second – *P. nigra* and *P.* × *sibirica*, third – species from the section *Tacamahaca* and one *P. nigra* genotype (SRR3045931) (Supplementary Data 6). When the region of *ARR17* partial repeats (Chr18:16,290,253-16,308,089) was used for the analysis, *P. nigra* and *P. deltoides* formed two distant clusters again, but *Tacamahaca* genotypes were divided into two groups, one of which (comprising most *Tacamahaca* samples) was closer to the *P. deltoides* cluster than to the one that contained several *P. balsamifera* samples. *P.* × *sibirica* and one *P. balsamifera* genotype (SRR1821331) belonged to the *P. nigra* group (Supplementary Data 7). We also performed clustering based on the whole SDR region (Chr18:16,200,000-16,320,000). This type of analysis provided the clearest concordance between clusters and *Populus* species – *P. nigra*, *P. deltoides*, and *Tacamahaca* species (*P. trichocarpa*, *P. balsamifera*, *P. simonii*, *P. cathayana*, *P. koreana*) formed three distinct clusters (Figure 4). *P.* × *sibirica* belonged to the *P. nigra* group again that is consistent with the assumption of *P.* × *sibirica* origin from *P. nigra* (Mayorov et al., 2012; Kostina and Nasimovich, 2014) and indicates that the Y haplotype of the studied *P.* × *sibirica* was probably derived from *P. nigra*. Remarkably, within SDR polymorphisms, many species-specific ones were identified (Supplementary Data 8). For example, polymorphisms in coordinates 16,200,264, 16,200,310, 16,200,550, 16,200,813, 16,201,718, 16,201,908, 16,203,249, 16,203,398, 16,206,677, 16,207,057, 16,272,448, 16,272,453, 16,272,879, 16,272,899, 16,272,413 (Chromosome 18, *P. trichocarpa* “Stettler 14” genome) were specific to *P. nigra* and *P.* × *sibirica*; 16,206,619, 16,206,640, 16,206,703, 16,206,719, 16,206,812, 16,206,822, 16,206,945 *–* to *P. deltoides*; 16,200,813, 16,205,821 *–* to *P. nigra*, *P.* × *sibirica*, and *P. deltoides.* It is worth noting that *P. cathayana* SRR9007071, *P. simonii* SRR9007072, *P. koreana* SRR12235434, and *P. balsamifera* SRR1821319 had a significant number of polymorphisms that were common to *P. nigra* and *P. deltoides* but not the majority of *P. trichocarpa* and *P. balsamifera* samples. Nevertheless, these four samples clustered with other species of the section *Tacamahaca* based on the complete SDR sequence.

**FIGURE 5:**
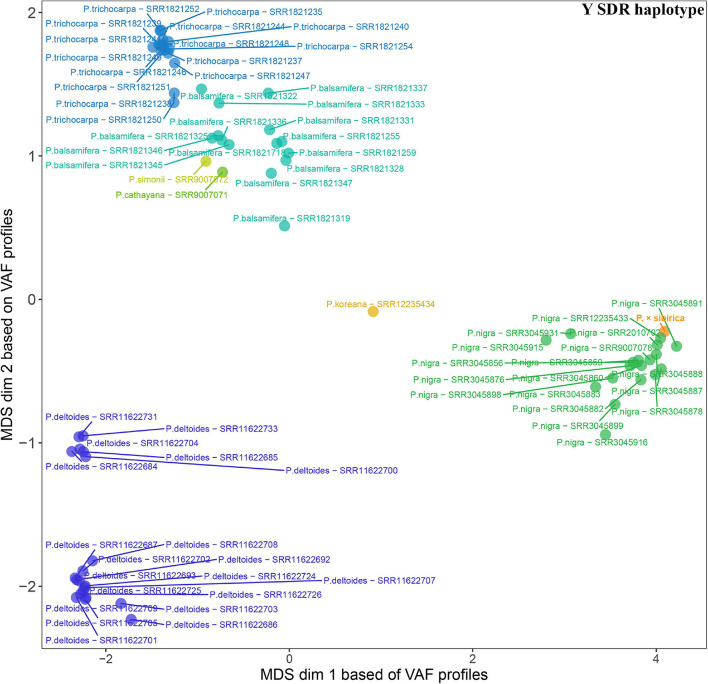
MDS plot for 70 *Populus* males and the male *P.* × *sibirica* based on Illumina WGS data aligned against the male *P.* × *sibirica* genome assembly with a further search for polymorphisms in the Y SDR haplotype.

**FIGURE 6:**
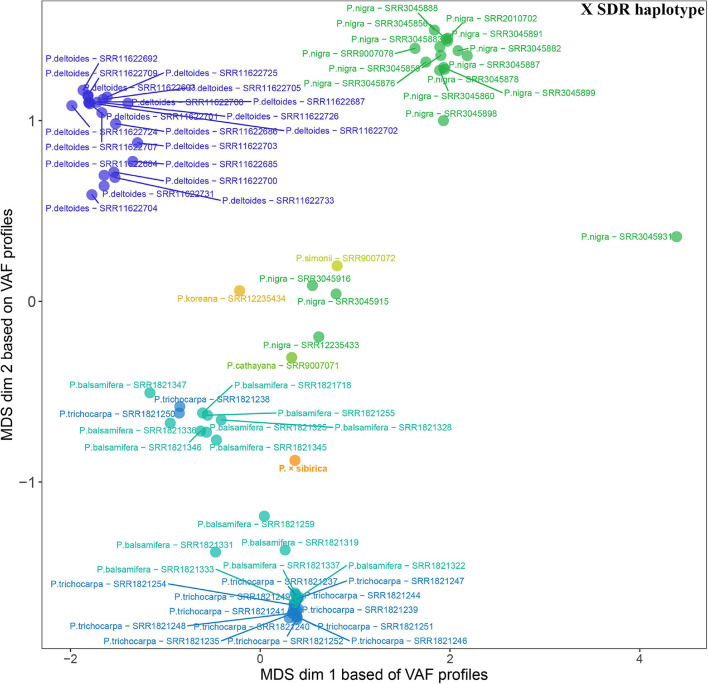
MDS plot for 70 *Populus* males and the male *P.* × *sibirica* based on Illumina WGS data aligned against the male *P.* × *sibirica* genome assembly with a further search for polymorphisms in the X SDR haplotype.

Given that SDR haplotypes of the examined *P.* × *sibirica* individuals differed significantly by the presence of long regions (up to several kb) with insertions/deletions and numerous SNPs, we supposed that the alignment of Illumina reads of *Populus* species against the male *P.* × *sibirica* assembly with two SDR haplotypes can give additional information on the relationship of *P.* × *sibirica* with other poplars. To investigate this issue, we aligned Illumina WGS data for the male *P.* × *sibirica* and 70 males of poplar species listed above against the male *P.* × *sibirica* genome assembly, identified polymorphisms in the Y [tig00001299, from 1,090,323 to the end of contig (1,200,738)] and the X [tig00000650, from 1,585,216 to the end of contig (1,676,203)] haplotypes of the SDR, and built MDS plots (Figures 5, 6) and dendrograms (Supplementary Data 9, 10). Based on the Y SDR haplotype, *P.* × *sibirica* formed clusters with *P. nigra* samples (Figure 5 and Supplementary Data 9), however, based on the X SDR haplotype, *P.* × *sibirica* clustered within *P. balsamifera* and *P. trichocarpa* group (Figure 6 and Supplementary Data 10). Thus, the performed analysis brought novel results and allowed us to suggest origins for both SDR haplotypes of *P.* × *sibirica*. Besides, differences in clusterization of *Populus* species were revealed in the MDS plots based on the separate Y and X SDR haplotypes. For the Y SDR haplotype (from tig00001299), two groups of *P. deltoides* were formed, *P. simonii* and *P. cathayana* were close to the group of *P. trichocarpa* and *P. balsamifera*, while *P. koreana* was located separately from all the other samples (Figure 5). For the X SDR haplotype (from tig00000650), *P. balsamifera* and *P. trichocarpa* samples formed two groups – the first one included most *P. trichocarpa* and a half of *P. balsamifera* samples, and the second one comprised the other half of *P. balsamifera* and several *P. trichocarpa* samples. *P. simonii*, *P. koreana*, and to a lesser extent *P. cathayana* individuals were separated from *P. balsamifera* and *P. trichocarpa* groups. Surprisingly, three *P. nigra* samples were closer to *P. balsamifera* and *P. trichocarpa* groups than to the main *P. nigra* cluster (Figure 6). It is worth noting that there were regions in the SDR that were specific to particular *Populus* species (Supplementary Data 11, 12).

### Identification of Repeats in the SDR

During the analysis of the SDR of *P.* × *sibirica* and related *Populus* species by the alignment of Illumina WGS data to the SDR of male *P. trichocarpa* “Stettler 14” (Chr18:16,200,000-16,320,000; 120 kb), we noticed regions with significantly increased coverage (up to 100-fold), including Chr18:16,280,032-16,289,301 (9.3 kb, see Supplementary Data 13). This locus was identified in the Y SDR haplotype of *P.* × *sibirica* but was absent in three X SDR haplotypes. It was located between the *ARR17* partial repeats (present only in the Y SDR haplotype of *P.* × *sibirica* and related poplars) and the *TCP* gene (present in both Y and X SDR haplotypes). When analyzing the Illumina read coverage profiles (Supplementary Data 13), it can be seen that for the half of the locus (Chr18:16,284,417-16,289,301; 4.8 kb) the coverage remains high along the entire length, while in the other half of the locus (Chr18:16,280,032-16,284,418; 4.4 kb) there are small regions with low coverage which is similar to the coverage of most of the SDR. The blastn analysis across genome assemblies of *Populus* species showed that there are homologues of two parts of the 9.3 kb locus (from about 0.5 to 3.0 kb and about 4.5 to 9.0 kb in the locus), which numbered dozens and hundreds of copies in genome assemblies of *P.* × *sibirica* (both male and female), *P. trichocarpa* (Phytozome: “Stettler 14”; NCBI: GCF_000002775.4), *P. deltoides* (NCBI: GCA_014884945.1 and GCA_014885025.1), *P. simonii* (NCBI: GCA_007827005.2), *P. davidiana* (NCBI: GCA_014885075.1), *P. euphratica* (NCBI: GCA_000495115.1), *P. ilicifolia* (NCBI: GCA_015708895.1), *P. alba* (NCBI: GCA_005239225.1), and *P. tremula* (PlantGenIE^4^ : W52). However, in other plant species, we did not find sufficiently long regions with homology to the locus (blastn search across all species except the genus *Populus*). Between the first (0.5–3.0 kb) and the second (4.5–9.0 kb) parts of the locus, there was a variable area; and in some cases, homologues only of the second part were found. Homologues of the 9.3 kb locus were revealed close to *ARR17* partial repeats in genome assemblies of males of *P.* × *sibirica*, *P. trichocarpa* (Phytozome: “Stettler 14”), *P. deltoides* (NCBI: GCA_014884945.1), *P. simonii* (NCBI: GCA_007827005.2), *P. davidiana* (NCBI: GCA_014885075.1), and *P. tremula* (PlantGenIE (see text footnote 4): W52). Moreover, the homologues were also identified 15-40 kb upstream of the *ARR17* gene in the genomes of *P.* × *sibirica* (both male and female), *P. trichocarpa* (Phytozome: “Stettler 14”; NCBI: GCF_000002775.4), *P. deltoides* (NCBI: GCA_014884945.1 and GCA_014885025.1), *P. simonii* (NCBI: GCA_007827005.2), *P. alba* (NCBI: GCA_005239225.1), and *P. tremula* (PlantGenIE (see text footnote 4): W52).

We performed the search for ORFs in this 9.3 kb locus, and five ORFs encoding proteins of at least 100 a.a. were revealed. For one of the ORFs (Chr18:16,286,214-16,287,161, reverse), Transposase_21 (InterPro entry IPR004242, Pfam entry PF02992) was identified, while for another one (Chr18:16,288,009-16,288,371, reverse), Transposase-associated domain (IPR029480 and PF13963) was revealed.

**FIGURE 7:**
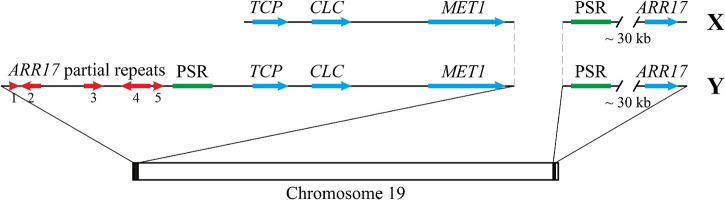
The scheme representing the SDR and *ARR17* gene region in male and female *P.* × *sibirica*. PSR – *Populus*-specific repeat.

Since (1) two parts of the 9.3 kb locus (about 0.5–3.0 and 4.5–9.0 kb) were found based on the Illumina read coverage and blastn analyses, (2) variable sequences were present between these two parts, (3) homologues of the second part were present in *Populus* genome assemblies not only in tandem with homologues of the first part but also separately, and (4) transposase domains were identified in the second part, we suggested that the second part of the locus (Chr18:16,284,417-16,289,301) is more important and deserves a further study. Thus, at the border of the common and unique parts of Y and X haplotypes of the SDR of poplars related to *P.* × *sibirica* (sections *Aigeiros* and *Tacamahaca*), we found a 5 kb repeat that contains a transposase-encoding ORF. This *Populus*-specific repeat (PSR) was also located near *ARR17* partial repeats in *P. tremula* and *P. davidiana* and was present near the *ARR17* gene in a number of *Populus* species. The scheme of the SDR and *ARR17* gene region in male and female *P.* × *sibirica* is presented in Figure 7.

## Discussion

Obtaining high-quality genome assemblies is an important step for the identification of genotype-specific differences at the whole genome level, therefore, the third-generation sequencing methods opened up novel opportunities for research in plant genomics (Li et al., 2017; Li and Harkess, 2018; van Dijk et al., 2018). The latest studies of *Salicaceae* are a good example of how using long reads for genome assembly can reveal a significant diversity of SDR location and nucleotide sequences in different species of the same family and identify sex-associated differences (Müller et al., 2020; Xue et al., 2020; Yang et al., 2020; Zhou et al., 2020a, b; He et al., 2021). However, long-read sequencing is very sensitive to the purity and length of input DNA, and for plant samples rich in diverse secondary metabolites, including polysaccharides and phenols, the use of an effective for a particular species method of extraction is necessary. Therefore, in the present study, we have tested several approaches recommended for the isolation of pure DNA: DNeasy Plant Mini Kit (Qiagen), CTAB method and its modifications, additional purification of the isolated DNA on magnetic particles using the CleanMag DNA kits (Evrogen) and AMPure XP beads (Beckman Coulter), purification of DNA after agarose gel electrophoresis using the QIAEX II Gel Extraction Kit (Qiagen), additional purification step with the Blood and Cell Culture DNA Mini Kit (Qiagen) recommended by Oxford Nanopore Technologies for plant DNA^5^, and isolation of the high-molecular-weight DNA fraction with the Short Read Eliminator Kit (Circulomics). As a result, we developed an optimal protocol for pure high-molecular-weight DNA extraction from leaves of *P.* × *sibirica* that enabled us to perform genome sequencing on the ONT platform and obtain high genome coverage with long reads (62 × and 46 × coverage with N50 of more than 20 kb for the male and female, respectively) that was extremely important for the further genome assembly.

Performance of a genome assembler strongly depends on the length and complexity of a genome, number and length of reads, and other parameters; and the suitable tool should be adequately selected for an organism under study (Jayakumar and Sakakibara, 2019; Dmitriev et al., 2020; Vaser and Šikić, 2020; Dvorianinova et al., 2021). Therefore, assemblies of male and female *P.* × *sibirica* genomes were performed using five tools (Canu, Flye, Raven, Shasta, and wtdbg2), and the comparison of the results revealed that the best genome assemblies were achieved using Canu – this tool outperformed others in one of the most important parameters: the best contig contiguity (the highest N50 and the lowest L50 values, the greatest length of the largest contig), the highest BUSCO completeness, the largest fraction of reference genome covered, the greatest number of covered reference genomic “features” (i.e., exons, transcripts, genes). In terms of the set of parameters, the assembly of the female *P.* × *sibirica* genome was slightly worse than the assembly of the male one (Table 1), which can be explained by a lower coverage with Nanopore reads – the coverage of the female genome was a quarter lower than that of the male genome. Canu is one of the first assemblers developed for third-generation sequencing data (Koren et al., 2017) and requires much more CPU hours compared to later developed Flye, Raven, Shasta, and wtdbg2. However, Canu is the most commonly used tool for the assembly of *Populus* genomes to date (Müller et al., 2020; Xue et al., 2020; Yang et al., 2020; Zhou et al., 2020b).

The sizes of *Populus* genome assemblies are in a range of about 400–500 Mb (Tuskan et al., 2006; Hofmeister et al., 2020; Müller et al., 2020; Xue et al., 2020; Yang et al., 2020). So, we expected to get the same assembly length; however, both our *P.* × *sibirica* assemblies were significantly longer – about 800 Mb. Along with this, we observed a high proportion of duplicated BUSCO. Besides, self-alignment of genomes revealed that only about 15% of the male and female *P.* × *sibirica* genome assemblies aligned against themselves one time and about 45% aligned against themselves two times. Thus, it became clear that we obtained the assemblies with abundantly separated haplotypes. We decided not to purge them [for example, with the Purge Haplotigs tool (Roach et al., 2018)] and keep the data for both haplotypes of the SDR and *ARR17* gene, which are known to play a key role in the *Populus* sex determination. To increase the assembly accuracy, we performed consistent polishing of genome assemblies according to the previously optimized scheme – polishing with ONT reads using Racon (twice) and Medaka and then with Illumina reads using POLCA (Dmitriev et al., 2020). Polishing resulted in the increase in BUSCO completeness from 97.71 to 98.45% for the male genome and from 96.53 to 98.20% for the female one, which are high values indicating that the obtained assemblies are complete enough. For example, BUSCO completeness for the genome of *P. trichocarpa* “Stettler 14” is 97.96%. Noticeably, the percentage of duplicated BUSCO increased drastically after polishing *P.* × *sibirica* genome assemblies – from 65.61 to 87.48% for the male and from 61.96 to 76.77% for the female.

Illumina reads are high-precision but short, so, their use for assembly polishing can result in wrong corrections with reads from other repeat instances^6^. However, in our case, less than 0.2% of the Illumina reads were mapped by BWA to multiple genomic loci for the male and female *P.* × *sibirica* assemblies. *P.* × *sibirica* is an intersectional hybrid between poplars from sections *Aigeiros* (*P. nigra*) and *Tacamahaca* (probably, *P. laurifolia* and *P. suaveolens*) (Mayorov et al., 2012; Kostina and Nasimovich, 2014; Kostina et al., 2017), therefore, the difference between its haplotypes was likely significant enough for polishing, and using accurate but short Illumina reads did not unify the haplotypes but improved the quality of *P.* × *sibirica* genome assemblies. This enabled us to perform a more detailed and careful analysis of the SDR variants in both male and female genomes of *P.* × *sibirica*.

Two contigs carrying the SDR were identified in both male (tig00001299 and tig00000650) and female (tig00001482 and tig00003220) genome assemblies of *P.* × *sibirica.* In tig00001299, partial repeats of the *ARR17* gene were present. Previous studies showed that in the Y SDR variant, inverted repeats of *ARR17* are present and produce small RNAs, which leads to silencing of the *ARR17* gene and plays a key role in sex determination of poplars and aspens (Müller et al., 2020; Xue et al., 2020; Yang et al., 2020; Zhou et al., 2020b). Thus, tig00001299 of *P.* × *sibirica* included the Y SDR haplotype, while tig00000650, tig00001482, and tig00003220 with no *ARR17* partial repeats included the X SDR haplotypes (Figure 2). We also identified a 5 kb repeat that was located between *ARR17* partial repeats and the *TCP* gene in the Y SDR variant of *P.* × *sibirica* and poplars from sections *Aigeiros* and *Tacamahaca*. This 5 kb repeat had homology with a significant number of genome regions of *Populus* species (but not other species), including that of the *ARR17* gene, and contained ORFs encoding transposase domains. In studies on *P. trichocarpa* and *P. deltoides*, *Gypsy* and *Copia* long terminal repeat (LTR) retrotransposons were identified in the SDR, and, in the male-specific region, fragments of *Gypsy* LTR were predominantly present (Xue et al., 2020; Zhou et al., 2020b). In the study of Yang et al., *Helitron-like* transposons and/or *Copia-like* LTR were revealed in the SDR and/or near the *ARR17* gene(s) (referred to as *RR*) in *P. euphratica*, *P. alba*, and *P. trichocarpa*. In the SDR of *P. euphratica*, *Helitron* transposons were located upstream of five out of six small *ARR17* partial repeats (referred to as partial *RR* duplicates), while a fragment of *Copia-like* LTR was located downstream of each of four large *ARR17* partial repeats. In the region of the *ARR17* gene(s) of *P. alba*, *P. euphratica*, and *P. trichocarpa*, a fragment of *Copia-like* LTR was revealed. Upstream of *ARR17* gene(s), *Helitron*-like elements were present in *P. alba* but not in *P. euphratica* and *P. trichocarpa.* As a result, it was suggested that *Helitron* transposons were involved in sex chromosome formation in poplars *via* capturing sequences of the *ARR17* gene (Yang et al., 2020). In the present study, we revealed the *Populus*-specific repeat (PSR), which is located close to *ARR17* partial repeats and upstream of the *ARR17* gene (Figure 7), and assume that it can be involved in translocation of the *ARR17* gene or its part to the SDR of *P.* × *sibirica* and other *Populus* species.

We also performed the assessment of similarity between the male *P.* × *sibirica* and the species from sections *Aigeiros* and *Tacamahaca* based on *ARR17* and SDR sequences by mapping Illumina reads against the two reference genomes: male *P. trichocarpa* “Stettler 14” and our assembly of male *P.* × *sibirica*, in which Y and X haplotypes of the SDR were present and differed significantly. The use of *P.* × *sibirica* genome assembly with separated haplotypes as a reference allowed us to determine the probable origin of Y and X haplotypes of the SDR of the studied *P.* × *sibirica*, which was closer to *P. nigra* based on the Y SDR haplotype but to *P. balsamifera* and *P. trichocarpa* based on the X SDR haplotype. Our results suggest that the use of genome assemblies with separated haplotypes can give additional information for phylogenetic analysis of poplars taking into account the presence of two diverse ancestors. This is especially important, as numerous studies of *Populus* phylogeny showed that a classification system based on morphological characteristics may differ from that based on molecular data (Hamzeh and Dayanandan, 2004; Cervera et al., 2005; Wang et al., 2014; Zhou et al., 2018; Zhang et al., 2019). Therefore, the analysis of the *Populus* SDR in addition to chloroplast, ITS (internal transcribed spacer), and other sequences traditionally used in the phylogenetic analysis could establish the origin and evolution of poplars, especially in the case of interspecific *Populus* hybrids. Besides, studies of the SDR of different *Populus* species are necessary for the development of molecular markers for sex identification that could be of help in city landscaping with only male poplars, thus, finding a solution to the problem of fluff.

## Data Availability Statement

The obtained sequencing data and genome assemblies of male and female *P.* × *sibirica* individuals can be found in the NCBI database under the BioProject accession number PRJNA644206.

## Author Contributions

NM and AD conceived and designed the work. NM, EP, AB, RN, LP, NB, AS, and AD performed the experiments. NM, EP, ED, AK, GK, and AD analyzed the data. NM, EP, ED, GK, and AD wrote the manuscript. All authors read and approved the final manuscript.

## Conflict of Interest

The authors declare that the research was conducted in the absence of any commercial or financial relationships that could be construed as a potential conflict of interest.

## Publisher’s Note

All claims expressed in this article are solely those of the authors and do not necessarily represent those of their affiliated organizations, or those of the publisher, the editors and the reviewers. Any product that may be evaluated in this article, or claim that may be made by its manufacturer, is not guaranteed or endorsed by the publisher.

## Footnotes

1
https://github.com/rrwick/Porechop

2
https://phytozome-next.jgi.doe.gov/info/PtrichocarpaStettler14_v1_1

3
https://github.com/nanoporetech/medaka

4
ftp://plantgenie.org/Publications/Muller2019/

5
https://community.nanoporetech.com/extraction_methods

6
https://canu.readthedocs.io/en/latest/faq.html

## Supplementary Material

Supplementary Data 1 Partial sequences of the *ARR17* gene with an upstream region and five *ARR17* partial repeats of males of *P*. × *sibirica*, *P. trichocarpa*, *P. deltoides*, and *P. simonii* used for the phylogenetic analysis. In the *P.* × *sibirica* genome assembly, two allelic variants of the *ARR17* gene were identified [designated *ARR17* (c1) and *ARR17* (c2)]. *ARR17* partial repeat 1 is the closest to the end of the chromosome and the subsequent ones are located further from the end in ascending order, according to the *P. trichocarpa* “Stettler 14” genome assembly.

Supplementary Data 2 Self-alignment of the male and female *P.* × *sibirica* assemblies and genomes of other *Populus* species from the NCBI database.

Supplementary Data 3 Sequences of four contigs that contain SDR variants of the male (tig00001299 – Y haplotype, tig00000650 – X haplotype) and female (tig00001482 and tig00003220 – X haplotypes) *P.* × *sibirica*. The sequences are presented in separate files in the fasta format.

Supplementary Data 4 Clusterization of the *TCP*, *CLC*, and *MET1* genes of the male (tig00001299 – Y haplotype, tig00000650 – X haplotype) and female (tig00001482 – X haplotype and tig00003220 – X haplotype) *P.* × *sibirica*.

Supplementary Data 5 Clusterization of the *ARR17* gene and five *ARR17* partial repeats of males of *P*. × *sibirica*, *P. trichocarpa*, *P. deltoides*, and *P. simonii*. In the *P.* × *sibirica* genome assembly, two allelic variants of the *ARR17* gene were identified [designated *ARR17* (c1) and *ARR17* (c2)]. *ARR17* partial repeat 1 is the closest to the end of the chromosome and the subsequent ones are located further from the end in ascending order, according to the *P. trichocarpa* “Stettler 14” genome assembly.

Supplementary Data 6 Clusterization of 70 *Populus* males and the male *P.* × *sibirica* based on Illumina WGS data aligned to the male *P. trichocarpa* “Stettler 14” genome with a further search for polymorphisms in the *ARR17* gene.

Supplementary Data 7 Clusterization of 70 *Populus* males and the male *P.* × *sibirica* based on Illumina WGS data aligned to the male *P. trichocarpa* “Stettler 14” genome with a further search for polymorphisms in the region of *ARR17* partial repeats.

Supplementary Data 8 Genetic polymorphisms of *P.* × *sibirica*, *P. nigra*, *P. deltoides*, *P. trichocarpa*, *P. balsamifera*, *P. simonii*, *P. cathayana*, *P. koreana* males based on Illumina WGS data aligned to the male *P. trichocarpa* “Stettler 14” genome with a further search for polymorphisms in the SDR. The numbers located one below the other indicate the number of reads with the reference (bottom) and alternative (top) alleles.

Supplementary Data 9 Clusterization of 70 *Populus* males and the male *P.* × *sibirica* based on Illumina WGS data aligned to the male *P.* × *sibirica* genome assembly with a further search for polymorphisms in the Y SDR haplotype.

Supplementary Data 10 Clusterization of 70 *Populus* males and the male *P.* × *sibirica* based on Illumina WGS data aligned to the male *P.* × *sibirica* genome assembly with a further search for polymorphisms in the X SDR haplotype.

Supplementary Data 11 Genetic polymorphisms of *P.* × *sibirica*, *P. nigra*, *P. deltoides*, *P. trichocarpa*, *P. balsamifera*, *P. simonii*, *P. cathayana*, *P. koreana* males based on Illumina WGS data aligned to the male *P.* × *sibirica* genome assembly with a further search for polymorphisms in the Y SDR haplotype. The numbers located one below the other indicate the number of reads with the reference (bottom) and alternative (top) alleles.

Supplementary Data 12 Genetic polymorphisms of *P.* × *sibirica*, *P. nigra*, *P. deltoides*, *P. trichocarpa*, *P. balsamifera*, *P. simonii*, *P. cathayana*, *P. koreana* males based on Illumina WGS data aligned to the male *P.* × *sibirica* genome assembly with a further search for polymorphisms in the X SDR haplotype. The numbers located one below the other indicate the number of reads with the reference (bottom) and alternative (top) alleles.

Supplementary Data 13 Genetic polymorphisms of *P.* × *sibirica*, *P. nigra*, *P. deltoides*, *P. trichocarpa*, *P. balsamifera*, *P. simonii*, *P. cathayana*, *P. koreana* males based on Illumina WGS data aligned to the SDR of male *P. trichocarpa* “Stettler 14” with a further search for polymorphisms. Alt. cov. – number of reads with the reference allele, Ref. cov. – number of reads with the alternative allele.
