# Supplementary material for: Genome Assembly and Sex-Determining Region of Male and Female Populus × sibirica
Source: Front Plant Sci. 2021 Sep 8;12:625416. doi: 10.3389/fpls.2021.625416 (PMC8455832; doi:10.3389/fpls.2021.625416)
Supplement: Supplementary Data 1 — Partial sequences of the ARR17 gene with an upstream region and five ARR17 partial repeats of males of P. × sibirica, P. trichocarpa, P. deltoides, and P. simonii used for the phylogenetic analysis. In the P. × sibirica genome assembly, two allelic variants of the ARR17 gene were identified [designated ARR17 (c1) and ARR17 (c2)]. ARR17 partial repeat 1 is the closest to the end of the chromosome and the subsequent ones are located further from the end in ascending order, according to the P. trichocarpa “Stettler 14” genome assembly. [file Data_Sheet_1.PDF]

Supplementary Data 1. Partial sequences of the *ARR17* gene with an upstream region and five *ARR17* partial repeats of males of *P.* × *sibirica*, *P. trichocarpa*, *P. deltoides*, and *P. simonii* used for the phylogenetic analysis. In the *P.* × *sibirica* genome assembly, two allelic variants of the *ARR17* gene were identified (designated *ARR17* (c1) and *ARR17* (c2)). *ARR17* partial repeat 1 is the closest to the end of the chromosome and the subsequent ones are located further from the end in ascending order, according to the *P. trichocarpa* "Stettler 14" genome assembly.

>*P.* × *sibirica ARR17* (c1)
ATAGTAAACAAATCCTAATAAAATCATGAGTCAGAGATAATTGCTAATCTTTTCAG
TCTTTGGGATTTGATTGCAGAAGCAGTAAAAGCAAAAAGATACCTTCTTGTTTTTGT
TTTTCCCTTTCTTCAAGAGATCTCCATGCTCAAAACCATTGTATATTTTAACACACT
TGTACGTACTTCCTATTCCATCTCCACATAGACAACACACGGTTGTTCAAATATTAT
TCCTTCCTACCTTACTTATAGGGCTTCAGATATGCAATCCATGCATGTCCATTCATT
TTTCTTTTACCCCTTCTTTGGAATCTTAATTACACCACCCCACATCACAAAAGTATT
AACTTAATTTTAAAAATAAAAGAATATAATAAATCTCTTTAATCACAAGGCCAATC
CTTTCATATTTGTAACCTATATCTATGTACCATGTAACATAGTACTATATTAGTAAC
ACAATATTAATATTTGTGGAGTGAGTTGATGACAAATTGTACTGTTGTTGTCTTAGA
AAGAGGTCTCATTTCTATATATTACCAATTTGCTTGTAAGA
>*P.* × *sibirica ARR17* (c2)
ATAGTAAACAAATCCTAATAAAATCATGAGTCAGAGATAATTGCTAATCTTTTCAG
TCTTTGGGATTTGATTGCAGAAGCAGTAAAAGCAAAAGGATACCTTCTTGTTTTTGT
TTTTCCCTTTCTTTAAGAGATTTCCATGCTCAAAACCATTGTATATTTTAACACACCT
GTACGTACTTCTTATTCCATCTCCACAGAGACAACACACGGTTGTTCAAATATTATT
CTTTCCTGCCTTACTTTTAAGGCTTCAGATATGCATTCCATGCATGTCCATTCATTTT
TCTTTTACCCCTTTTTTAGAATCTTAATTACACCACCCCACATCACAAAAGTATTAA
CTTAATTTTAAAAATAAAAGAATATAATAAATCTCTTTAATCACAAGGCCAATCCT
TTCAGATTTGTAACCTAGATCTATGTACCACGTAACATAGTACTATATTAGTAACAC
AATATTAAAATTTTTAGAGTGAGTTGATGACAAATTGTACTGTTGTTGTCTTAGAAG
GACGTCTCATTTCTATACATTACCAATTTGCTTGTAAAA
>*P.* × *sibirica ARR17* repeat 1
ATAGTAAATAAATCCTAATAAAATCATGAGTTGGAGATAATTGCTGATCTTTTTAG
TCTTTGGGATTTGATTGCAGAAGCAGTAAAAACGAAATAGATACCTTGTTTTTGTTT
TTCCCTTTCTTCAAGTGATCTCCATGCTCAAAACCATTGTATATTTTAACACACTTG
TACGTACTTCCTATTCCATCTCCACACAGACAATACACGGTTATTCAAATATTATGT
ATTCCTTCCTGCCTTACTTGTAGGGCTTCGGATATGCATTCCATGCATGTCCATTCA
TTTTTCTTTTACCCTTTCTTTAGAATCTTAATTACATCACCCCACATCATAAAAGTAT
TAACTTAATTTTAAAAATAAAATAATATAATAAATCACTTTAATCACAAGACCAAT
CCTTTCAGATTTGTAACCTAAATCTATTTACCACGTAACATAGTACTATATTAGTAA
TACATATAATATTAATATTTATGGAGTGAATTGATAACAAATGGTACTGTTGTTGCC
TTAGAAAGAGGTCTCATTTCTATAAATTACCAATTTGCTTGCAAGA
>*P.* × *sibirica ARR17* repeat 2
ATAGTAAACAAATCCTAATAAAATCATGAGTCAAAGATAATTGCTGATTTTTTCGG
TCTTTAGGATTTGATTGCAGAAGCAGTAAAAGCGAAATAGATACCTTCTTATTTTTG
TGTTTCCCTTTTTTCAAGAGATCTGCATGCTCAAAACCATTGTATATTTTAACACAC
TTGTATGTACTTTCTATTCCGTCTCCACACAGACAACACACGGTTGTTCAAATATTA
TATATTCCTTCCTGCCTTACTTGTAGGGCTTTGGATGTGCATTCCATGCATGTCCATT
CATTTTTCTTTTACCCCTTCTTTAGAATCTTAATTACACCACCCCACATCATAAAAG
TCTTAATTTAATTTTAAAAATAAAAAAATATAATAAATCTCTTTAATCATAAACCA
ATCCTTTCATATTTGTAACCTAGATCCATGTACCACACAACATAGTAATATATTAAT
AACACATATGATATTAATATTTGTGGAGTGAGTTGATGACAAATGGTACTGTTGTT
GTCTTAGAAAGAAGCCTTATTTCTATACATTACCAATTTGCTTGTAAGA
>*P.* × *sibirica ARR17* repeat 3

ATAGTAAACAAATTCTAATAAAATCATGAGTCAGAGATAATTGTTGATCTTTTTGG
TCTTTAGGATTTGATTGCAGAAGCAGTAAAAGCGAAATAAATATTTTCTTGTTTTTG
TTTTTCCCTTTCTTCAAGAAATATCTATGCTCAAAACCATTGTATATTTTAACACAC
TTGTACGTACTTTGTATTCCATCTCCACACAAACAACACACAGTTGTTCAAATATTA
TACATTCCTTCCTGCCTTACTTGTAGGGCTTCGGATATGCATTCCATGCATGTTCAT
TCATTTTTCTTTTACCCCTTTTTTAGAATCTTAATTACACTACTCCACCTCACAAAAG
TATTAACTTAATTTTAAAAATAAAAGAATATAATAAATCTCTTTAATCACAAGACC
AATCCTTTTAGATTTGTAACCTAGATCTATGTACCACATAACATAGTACTATATTAG
TAATACACATAATATTAATATTTGTGGAGTGAGTTGATGACAAATGGTACTGTTGT
TGTCTTAGAAAGAGGTCTCATTTCTATACATTACCAATATGCTGTAAGA
>*P.* × *sibirica ARR17* repeat 4
ATAGTAAACAAATCCTAATAAAATCATGAGTCGAAGATAATTACTGATCTTTTCGG
CCTTTGGGTTTTGATTGCAGAAGTAGTAAAAGCGAAATAAATACCTTCTTATTTTTG
GTTTTACCTTTCTTCAAGAGATCTCCATGCTCAAAACCATTGTATATTTTAACACAC
TTGTATGTACTTCCTATTCCATCTCCACACAGAAAATACACGGTTGTTCAAATATTA
TATATTCCTTCCTGCCTTACTTGTAGGGCTTCGGATATGCATTCCATGCATGTCCAT
TCATTTTTCTTTTACCCCTTCTTTAGAATCTTAATTACACCACCCCACATCATAAAA
GTATTAAATTAATTTTAAAAATAAAAGAATATAATAAACCTATTAAATCACAAGAC
CAATCCTTTCATATTTGTAACCTCAATCTATGTACCACACAACATATTACTATATTA
GTAACACACACAATATTAATATTTGTGGAGTGAGTTGATGACAAATGGTATTATTA
TTGTCTTAGAAAGAGGTCTCATTTCTATAAATTACCAATTTGCTTGTAAGA
>*P.* × *sibirica ARR17* repeat 5
ATAATAAACAAAACCTAATAAAATCATGAGTCGAAGATAATTGCTGATCTTTTCGG
TCTTTGGGATTTGATTACAGAAGCAGTAAAAACGAAATACATACCTTCTTGTTTTTG
TTTTTCCCTTTCTTCAAGAGATCTCCATGCTCAAAACCATTGTATATTTTAACACAC
TTGTACGTACTTCCTATTCCATCTCCACACAGAAAACACATGGTTGTTCAAATATTA
TATATTCCTTTCTGCCTTACTTGTAGGGCTTCGGATATGCATTCCATGCATGTCCATT
CATTTTTCTTTTACCCCTTCTTTAGAATCTTAATTACACCACCCCACATCATAAAAG
TATTAACTTAATTTAAAAAATAAAAGAATATAATAAATGTCTTTAATCACAAGACC
AATCCTTTCAGATTTGTAACCTAGATCTGTGTACCACACAACATAGTACTATATTAG
TAACACACATAATATTAATATTTATGGAGTGAGTTGATGACAAATGGTATTGTTGT
TGTCTTAGAAAGAGGTCTCATTTCTATATATTACCAATTTGTTTGTAAGA

>*P. trichocarpa ARR17*
ATAGTAAACAAATCCTAATAAAATCATGAGTCAGAGATAATTGCTAATCTTTTCAG
TCTTTGGGATTTGATTGCAGAAGCAGTAAAAGCAAAAAGATACCTTATTGTTTTTG
TTTTTCCCTTTCTTCAAGAGATCTCCATGCTCAAAACCATTGTATATTTTAACACAC
TTGTACGTACTTCTTATTCCTTCTCCACAGAGACAACACACGGTTGTTCAAATATTA
TTCCTTCCTACCTTACTTGTAGGGCTTCAGATATGCAATCCATACATGTCCATTCAT
TTTTCTTTTACCCCTTCTTTGGAATCTTAATTACACCACCCCACATCACAAAAGTAG
TAACTTAATTTTAAAAATAAAAAAATATAATAAATCTCTTTAATCATAAGGCCAAT
CCTTTCAGATTTGTAACCTAGATCTATGTACCAAGTGACATAGTACTATATTAGTAA
CACAATATTAATATTTGTGGAGTGATAAATTGTACTGTTGTTGTCTTAGAAAGACGT
CTCATTTCTATATATTACCAATTTGCTTGTAAGA
>*P. trichocarpa ARR17* repeat 1
ATAGTAAATAAATCCTAATAAAATCATGAGTTGGAGATAATTGCTGATCTTTTTAG
TCTTTGGGATTTGATTGCAGAAGCAGTAAAAGCGAAATAGATACCTTGTTTTTGTTT
TTCCCTTTCTTCAAGTGATCTCCATGCTCAAAACCATTGTATATTTTAACACACTTG
TACGTACTTCCTATTCCATCTCCACACAGACAACACACGGTTATTCAAATATTATGT
ATTCCTTCCTGCCTTACTTGTAGGGCTTCGGATATGCATTCCATGCATGTCCATTCA
TTTTTCTTTTACCCCTTCTTTAGAATCTTAATTACAGCACCCCACATCATAAAAGTA
TTAACTTAATTTTAAAAATAAAATAATATAATAAATCACTTTAATCACAAGACCAA

TCCTTTTATGTTTGTAACCTATATCTATGTACCACATAACATAGTACTATATTAGTA
ACACACACAATATTAATATTTATGGAGTGAATTGATAACAAATGGTACTGTTGTTG
CCTTAGAAAGAGGTCTCATTTCTATAAATTACCAATTTGCTTGCAAGA
>*P. trichocarpa ARR17* repeat 2
ATAGTAAACAAATCCTAATAAAATCATGAGTCAAAGATAATTGCTGATCTTTTCGG
TCTTTAGGATTTGATTGCAGAAGCAGTAAAAGCGAAATAGATACCTTCTTATTTTTG
TGTTTCCCTTTTTTCAAGAGATCTGCATGCTCAAAACCATTGTATATTTTAACACAC
TTGTATGTACTTCCTATTCCGTCTCCACACAGACAACACACGGTTGTTCAAATATTA
TATATTCCTTCCTGCCTTACTTGTAGGGCTTCGGATATGCATTCCATGCATGTCCAT
TTATTTTTCTTTTACCTCTTCTTTAGAATCTTAATTACACCACCCCACGTCATAAAAG
TATTAACTTAATTTTAAAAATAAAAAAATATAATAAATCTCTTTAATCATAAGACC
AATCCTTTCATATTTGTAACCTAGATCCATGTACCACACAACATAGTACTATATTAA
TAACACACATGATATTAATATTTGTGGAGTGAGTTGATGACAAATGGTACTGTTGT
TGTCTTAGAAAGAGATCTCATTTCTATACATTACCAATTTGCTTGTAAGA
>*P. trichocarpa ARR17* repeat 3
ATAGTAAACAAATCCTAATAAAATCATGAGTCAGAGATAATTGCTGATCTTTTTGG
TCTTTAGGATTTGATTGCAGAAGCAGTAAAAGCGAAATAAATATTTTCTTGTTTTTG
TTTTTCCCTTTTTTCAAGAGATATCCATGCTCAAAACCATTGTATATTTTAACACAC
TTGTACGTACTTCGTATTCCATCTCCACACAAACAACACACAGTTGTTCAAATATTA
TACATTCCTTCCTGCCTTACTTGTAGGGCTTCGGATATGCATTCCATGCATGTCCAT
TCATTTTTCTTTTACCCCTTCTTTAGAATCTTAATTACACCACTCCACCTCACAAAAG
TATTAACTTAATTTTAAAAATAAAAGAATATAATAAATCTCTTTAATCACAAGACC
AATCCTTTCAGATTTGTAACCTAGATCTATGTACCACATAACATAGTACTATATTAG
TAATACACATAATATTAATATTTGTGGAGTGAGTTGATGACAAATGGTACTGTTGT
TGTTTTAGAAAGATGTCTCATTTCTATACATTACTAATTTGCTTGTAAGA
>*P. trichocarpa ARR17* repeat 4
ATAGTAAACAAATCCTAATAAAATCATGAGGCGGAGATAATTGCTAATCTTTTCGG
TCTTTGGGATTTGATTGCAGAAGAAGTAAAAGCGAAATAAATACCTTCTTATTTTT
GTTTTTACCTTTCTTCAAGAGATCTCCATGCTCAAAACCATTGTATATTTTAACACA
CTTGTACGTACTTCTTATTCCATCTCCACACAGAAAATACACGGTTGTTTAAATATT
ATATATTTCTTCCTGCCTTACTTGTAGGGCTTCGGATATGCATTCCATGCATGTCCA
TTCATTTTTCTTTTACCCCTTCTTTAGAATCTTAATTACACCACCCCACATCATAAAA
GTATTAAATTAATTTTAAAAATAAAAGAATATAATAAACCTATTTAATCACAAGAC
CAGTCCTTTCATATTTGTAACCTAAATCTATGTACCACACAACATATTACTATATTA
GTAACACACACAATATTAATATTTGTGGAGTGAGTTGATGACAGATGGTACTATTA
TTGTCTTAAAAAGAGGTCTCATTTCTATAAATTACCAATTTGCTTGTAAGA
>*P. trichocarpa ARR17* repeat 5
ATAGTAAACAAAACCTAATAAAATCATGAGTCGAAGATAATTGCTGATCTTTTCGG
TCTTTGGGATTTGATTACAGAAGCAGTAAAAACGAAATAAATATTTTCTTGTTTTTG
TTTTTCCCTTTCTTCAAGAGATCTCCATGCTCAAAACCATTGTATATTTTAACACAC
TTGTACGTACTTCCTATTCCATCTCCACACAGAAAGCACACGGTTGTTCAAATATTA
TATATTCCTTCCTGCCTTACTTGTAGGGCTTCGGATATGCATTCCATGCATGTCCAT
TCATTTTTCTTTTACCCCTTCTTTAGAATCTTAATTACACCACCCCGCATCATAAAA
GTATTAACTTAATTTTAAAAATAAAAGAATATAATAAATCTCTTTAATCACAAGAC
CAATCCTTTCAGATTTGTAACCTAGATCTATGTACCACACAACATAGTACTATATTA
GTAACACACATAATATTAATATTTGTGGAGTGAGTTGATGACAAATGGTATTGTTG
TTGTCTTAGAAAGAGGTCTCATTTCTATATAGTACCAATTTGTTTGTAAGA

>*P. deltoides ARR17*
ATAGTAAACAAATCCTAATAAAATCATGAGTCAGAGATAATTGCTAATCTTTTCAG
TCTTTGGGATTTGATTGCAGCAGCAGTAAAAGCAAAAAGATACCTTCTTGTTTTTGT
TTTTCCCTTTCTTCAAGAGATCTCCATGCTCAAAACCATTGTATATTTTAACACACT

TGTACGTACTTCCTATTCCATCTCCACAGAGACAACACACGGTTGTTCAAATATTAT
TCCTTCCTGCCTTACTTTTAGGGCTTCAGATATGCAATCCATGCATGTCCATTCATTT
TTCTTTTACCCCTTCTTTAGAATCTTAATTACACCACTCCACATCACAAAAGTATTA
ACTTAATTTTAAAAATAAAAGAATATAATAAATCTCTTTAATCACAAGGCCAATCC
TTTTAGATTTGTAACCTAGATCTATGTACCACGTAACATAGTACTATATTAGTAACA
CAATATTAATATTTGTGCAGTGAGTTGATGACAAATTGTACTGTTGTTGTTCTAGAA
AGAGGCCTCATTTCTATATATTACCAATTTGCTTGTAAGA
>*P. deltoides ARR17* repeat 1
ATAGTAGATAAATCCTAATAAAATCATGAGTTGGAGATAATTGCTGATCTTTTTAG
TCTTTGGGATTTGATTGCAGAAGCAGTAAAAGCGAAATAGATACCTTGTTTTTGTTT
TTCCCTTTCTTCAAGTGATCTCCATGCTCAAAACCATTGTATATTTTAACACACTTG
TACGTACTTCCTATTCCATCTCCATGTATTCCTTCCTGCCTTACTTGTAGGGCTTCGG
ATATGCATTCCATGCATGTCCATTCATTTTTCTTTTACCCCTTCTTTAGAATCTTAAT
TACAGCACCCCACATCATAAAAGTATTAACTTAATTTTAAAAATAAAATAATATAA
TAAATCACTTTAATCACAAGACCAATCCTTTCATGTTTGTAACCTACATCTATGTAC
CACATAACATAGTACTATATTAGTAACACACACAATATTAATATTTATGGAGTGAA
TTGATAACAAATGGTACTGTTGTTGCTTTAGAAAGAGGTCTCATTTCTATAAATTAC
CAATTTGCTTGCAAGA
>*P. deltoides ARR17* repeat 2
ATAGAAAACAAATCCTAATAAAATCATGAGTCAAAGATAATTGCTGATCTTTTCGG
TCTTTAGGATTTGATTGCAGAAGCAGTAAAAGCGAAATAGATACCTTATTTTTGTG
TTTCCCTTTTTTCAAGAGATCTGCATGCTCAAAACCATTGTATATTTTAACACACTT
GTATGTACTTCCTATTCCGTCTCCACACAGACAACACACGGTTGTTCAAATATTATA
TATTCCTTCCTGCCTTACTTGTAGGGCTTCGGATATGCATTCCATGCATGTCCATTT
ATTTTTCTTTTACCCCTTCTTTAGAATCTTAATTACACCACCCCACGTCATAAAAGT
ATTAACTTAATTTTAAAAATAAAAAAAATATAATAAATCTCTTTAATCATAAGACC
AATCCTTTCATATTTATAACCTAGATCCATGTACCACACAACATAGTACTATATTAA
TAACACACATGATATTAATATTTGTGGAGTGAGTTGATGACAAATGATACTGTTGT
TGTCTTAGAAAGAGATCTCATTTCTATACATTACCAATTTGCTTGTAAGA
>*P. deltoides ARR17* repeat 3
ATAGTAAACAAATCCTAATAAAATCATGAGTCAGAGATAATTGCTGATCTTTTTGG
TCTTTAGGATTTGATTGCAGAAGCAGTAAAAGCGAAATAAATATTTTCTTGTTTTTG
TTTTTCCCTTTTTTCAAGAGATATCCATGCTCAAAACCATTGTATATTTTAACACAC
TTGTACGTACTTCGTATTCCATCTCCACACAAACAACACACAGTTGTTCAAATATTA
TACATTCCTTCCTGCCTTACTTATAGGGCTTCGGATATGCATTCCATGCATGTCCAT
TCATTTTTCTTTTACCCCTTCTTTAGAATCTTAATTACACCACTCCACCTCACAAAAG
TATTAACTTAATTTTAAAAATAAAAGAATATAATAAATCTCTTTAATCACAAGACC
AATCCTTTCAGATTTGTAACCTAGATCTATGTACCACATAACATAGTACTATATTAG
TAATACACATAATATTAATATTTGTGGAGTGAGTTGATGACAAATGGTACTGTTGT
TGTTTTAGAAAGATGTCTCATTTCTATACATTACTAATTTGCTTGTAAGA
>*P. deltoides ARR17* repeat 4
ATAGTAAACAAATCCTAATAAAATCATGAGGCGGAGATAATTGCTAATCTTTTCGG
TCTTTGGGATTTGATTGCAGAAGAAGTAAAAGCGAAATAGATACCTTCTTATTTTT
GTTTTTACCTTTCTTCAAGAGATCTCCATGCTCAAAACCATTGTATATTTTAACACA
CTTGTACGTACTTCTTATTCCATCTCCACACAGAAAATACACGGTTGTTTAAATATT
ATATATTTCTTCCTGCCTTACCTCTAGGGCTTCGGATATGCATTCCATGCATGTCCA
TTCATTTTTCTTTTACCCCTTCTTTAGAATCTTAATTACATCACCCCACATCATAAAA
GTATTAAATTAATTTTAAAAATAAAAGAATATAATAAACCTATTTAATCACAAGAC
CAGTCCTTTCATATTTGTAACCTAAATCTATGTACTACACAACATATTACTATATTA
GTAACACACACAATATTAATATTTGTGGAGTGAGTTGATGACAGATGGTACTATTA
TTGTCTTAAAAAGAGGTCTCATTTCTATAAATTACCAATTTGCTTGTAAGA
>*P. deltoides ARR17* repeat 5

ATAGTAAACAAAACCTAATAAAATCATGAGTCGAAGATAATTGCTGATCTTTTCGG
TCTTTGGGATTTGATTACAGAAGCAGTAAAAACGAAATAAATATTTTCTTGTTTTTG
TTTTTCCCTTTCTTCAAGAAATCTCCATGCTCAAAACCATTGTATATTTTAACACAC
TTGTACGTACTTCCTATTCCATCTCCACACAGAAAGCACACGGTTGTTCAAATATTA
TATATTCCTTCCTGCCTTACTTGTAGGGCTTCGGATATGCATTCCATGCATGTCCAT
TCATTTTTCTTTTACCCCTTCTTTAGAATCTTAATTACACCACCCCGCATCATAAAA
GTATTAACTTAATTTTAAAAATAAAAGAATATAATAAATCTCTTTAATCACAAGAC
CAATCCTTTCAGATTTGTAACCTAGATCTATGTACCACACAACATAGTACTATATTA
GTAACACACATAATATTAATATTTGTGGAGTGAGTTGATGACAAATGGTATTGTTG
TTGTCTTAGAAAGAGGTCTCATTTCTATATAGTACCAATTTGTTTGTAAGA

>*P. simonii ARR17*
ATAGTAAACAAATCCTAATAAAATCATGAGTCAGAGATAATTGCTAATCTTTTCAG
TCTTTGGGATTTGATTGCAGAAGCAGTAAAAGCAAAAAGATACCTTCTTGTTTTTGT
TTTACCCTTTCTTCAAGAGATCTCCATGCTCAAAACCATTGTATATTTTAACACACT
TGTACGTAGTTCCTATTCCATCTCCACAGAGAACACACGGTTGTTCAAATATTATTC
CTACCTACCTTACTTGTAGGGCTTTAGATATGCAATCCATGCATGTACATTCATTTT
TCTTTTACCCTTCTTTGGAATCTTAATTACACCACCCCACATCACAAAAGTATTAAC
TTAATTTTAAAAATTAAAGAATATAATAAATCTCTTTAATCACAAGGCCAATCCTTT
CAGATTTGTAACCTAGATCTATGTACCAAGTAACATAGTACTATATTAGTCACACA
ATATTAATATTTGTGGAGTGAGTTGATGATAAATTGTACTATTGTTGTCTTAGAAAG
AGGTCTCATTTCTATATATTGCCAATTTGCTTGTAAGG

>*P. simonii ARR17* repeat 1
ATTGTAAATAAATCCTAATAAAATCATGAGTTGGAGATAATTGCTGATCTTTTTAGT
CTTTGGGATTTGATTGCAGAAGCAGTAAAAGCGAAATAGATACCTTGTTTTTGTTTT
TCCCTTTCTTCAAGTGATCTCCATGCTCAAAAGCATTGTATATTTTAACACACTTGT
ACGTACTTCCTATTCCATCTCCACACAGACAACACACGGTTATTCAAATATTATGTA
TTCCTTCCTGCCTTACTTGTAGGGCTTCGGATATGCATTCCATGCATGTCCATTCATT
TTTCTTTTACCCCTTCTTTAGAATCTTAATTACATCACCCCACATCATAAAAGTATT
AACTTAATTTTAAAAATAAAATAAAATAATAAATCACTTTAATCACAAGGCCAATC
CTTTCATATTTGTAACCTACATCTATGTACCACATAACATAGTACTATATTAGTAAC
ACACACAATATTAATATTTATGGAATGAATTGATAACAAATGGTACTGTTGTTGCC
TTAGAAAGAGGTCTCATTTCTATAAATTACCAATTTGCTTGCAAGA

>*P. simonii ARR17* repeat 2
ATAGTAAACAAATCCTAATAAAATCATGAGTCAAAGATAATTGCTGATCTTTTCGG
TCTTTAGGATTTGATTGCAGAAGCAGTAAAAGCAAAATAGATACCTTCTTATTTTA
GTGTTTCCCTTTTTTCAAGAGATCTGCAGCTCAAAACCATTGTATATTTTAACACAC
TTGTATGTACTTCCTATTCCGTCTCCACACAGACAACACACGGTTGTTCAAATATTA
TATATTCCTTCCTGCCTTACTTGTAGGGCTTCGGATATGCATTCCATGCATGTCCAT
TTATTTTTCTTTTACCCCTTCTTTAGAATCTTAATTACATCACCCCACGTCCTAAAAG
TATTAACTTAATTTTAAAAATAAAAAAATATAATAAATCTCTTTAATCATAAGACC
AATCCTTTCATATTTGTAACCTAGATCCATGTACCACACAACATAGTACTATATTAA
TAACACACATGATATTAATATTTGTGGAGTGAGTTGATGACAAATGGTACTGTTGT
TGTCTTAGAAAGAGATCTCATTTCTATACATTACCAATTTGCTTGTAAGA

>*P. simonii ARR17* repeat 3
ATAGTAAACAAATCCTAATAAAATCATGAGTCAGAGATAATTGCTGATCTTTTTGG
TCTTTAGGATTTGATTGCAGAAGCAGTAAAAGCGAAATAAATATTTTCTTGTTTTTG
TTTTTCCCTTTCTTCAAGAGATATCCATGCTCAAAACCATTGTATATTTTAACACAC
TTGTACGTACTTCGTATTCCATCTCCACACAAACAACACACAGTTGTTCAAATATTA
TACATTCCTTCCTGCCTTACTTGTAGGGCTTCGGATATGCATTCCATGCATGTCCAT
TCATTTTTCTTTTACCCCTTCTTTAGAATCTTAATTACACCACTCCACCTCACAAAAG
TATTAACTTAATTTTAAAAATAAAAGAATATAATAAATCTCTTTAATCACAAGACC

AATCCTTTCAGATTTGTAACCTAGATCTATGTACCACATAACATAGTACTATATTAG
TAATACACATAATATTAATATTTGTGGAGTGAGTTGATGACAAATGGTACTGTTGT
TGTTTTAGAAAGATGTCTCATTTCTATACATTACTAATTTGCTTGTAAGA
>*P. simonii ARR17* repeat 4
ATAGTAAACAAATCCTAATAAAATCATGAGTCGGAGATAATTGCTAATCTTTTCGG
TCTTTGGGATTTGATTGCAGAAGAAGTAAAAGCGAAATAGATACCTTCTTATTTTT
GTTTTTACCTTTCTTCAAGAGATCTCCATGCTCAAAGCCATTGTATATTTTAACACA
CTTGTACGTACTTCTTATTCCATCTCCACACAGAAAATACACGGTTGTTTAAATATT
ATATATTTCTTCCTGCCTTACTTGTAGGGCTTCGGATATGCATTCCATGCATGTCCA
TTCATTTTTCTTTTACCCCTTCTTTAGAATCTTAATTACACCACCCCACATCATAAAA
GTATTAAATTAATTTTAAAAATAAAAGAATATAATAAACCTATTTAATCACAAGAC
CAATCCTTTCATATTTGTAACCTAAATCTATGTACCACACAACATATTACTATATTA
GTAACACACACAATATTAATATTTGTGAAGTGAGTTGATGACAGATGGTACTATTA
TTGTCTTAAAAAGAGGTCTCATTTCTATAAATTACCAATTTGCTTGTAAGA
>*P. simonii ARR17* repeat 5
ATAGTAAACAAAACCTAATAAAATCATGAGTCGAAGATAATTGCTGATCTTTTCAG
TTTTTGGGATTTGATTACAGAAGCAGTAAAAACGAAATAGATACCTTCTTATTTTTG
TTTTTCCCTTTCTTCAAGAGATCTCCATGCTCAAAACCATTGTATATTTTAACACAC
TTGTACGTACTTCCTATTCCATCTCCACACAGAAAAAACACGGTTGTTCAAATATTA
TATATTCCTTCCTGCCTTACTTGTAGGGCTTCGGATATGCATTCCATGCATGTCCAT
TCATTTTTATTTTACCCCTTCTTTAGAATCTTAATTACACCACCCCACATCATAAAA
GTATTAACTTAATTTTAAAAATAAAAGAATATAATAAATCTCTTTAATCACAAGAC
CAATCCTTTCAGATTTGTAACCTAGATCTATGTACCATACAACATAGTACTATATTA
GTAACACACATAATATTAATATTTGTGGAGTGAGTTGATGACAAATGGTATTGTTG
TTGTCTTAGAAAGAGGTCTCATTTCTATATATTGCCAATTTGTTTGTAAGA
