# Supplementary material for: Genome Assembly and Sex-Determining Region of Male and Female Populus × sibirica
Source: Front Plant Sci. 2021 Sep 8;12:625416. doi: 10.3389/fpls.2021.625416 (PMC8455832; doi:10.3389/fpls.2021.625416)
Supplement: Supplementary Data 2 — Self-alignment of the male and female P. × sibirica assemblies and genomes of other Populus species from the NCBI database. [file Data_Sheet_2.PDF]

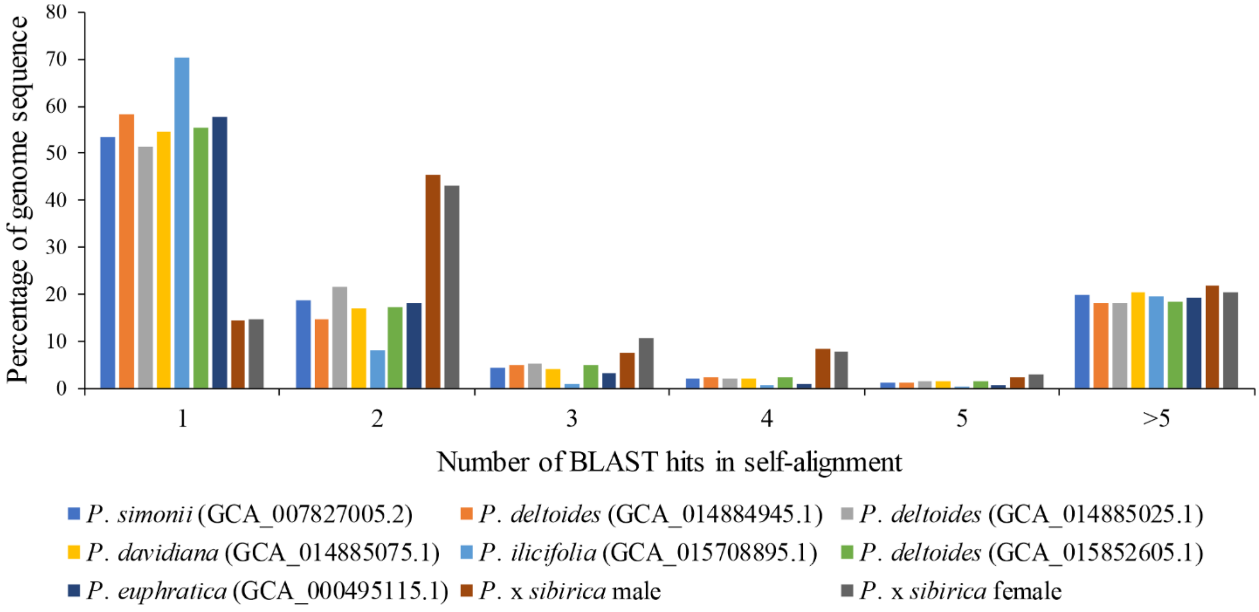

Supplementary Data 2. Self-alignment of the male and female *P.* × *sibirica* assemblies and genomes of other *Populus* species from the NCBI database.
