# Supplementary material for: Genome Assembly and Sex-Determining Region of Male and Female Populus × sibirica
Source: Front Plant Sci. 2021 Sep 8;12:625416. doi: 10.3389/fpls.2021.625416 (PMC8455832; doi:10.3389/fpls.2021.625416)
Supplement: Supplementary Data 3 — Sequences of four contigs that contain SDR variants of the male (tig00001299 – Y haplotype, tig00000650 – X haplotype) and female (tig00001482 and tig00003220 – X haplotypes) P. × sibirica. The sequences are presented in separate files in the fasta format. [file Data_Sheet_3.ZIP › Supplementary Data 3.pdf]

Supplementary Data 3. Sequences of four contigs that contain SDR variants of the male (tig00001299 – Y haplotype, tig00000650 – X haplotype) and female (tig00001482 and tig00003220 – X haplotypes) *P.* × *sibirica*. The sequences are presented in separate files in the fasta format.
