# Supplementary material for: Genome Assembly and Sex-Determining Region of Male and Female Populus × sibirica
Source: Front Plant Sci. 2021 Sep 8;12:625416. doi: 10.3389/fpls.2021.625416 (PMC8455832; doi:10.3389/fpls.2021.625416)
Supplement: Supplementary Data 4 — Clusterization of the TCP, CLC, and MET1 genes of the male (tig00001299 – Y haplotype, tig00000650 – X haplotype) and female (tig00001482 – X haplotype and tig00003220 – X haplotype) P. × sibirica. [file Data_Sheet_4.PDF]

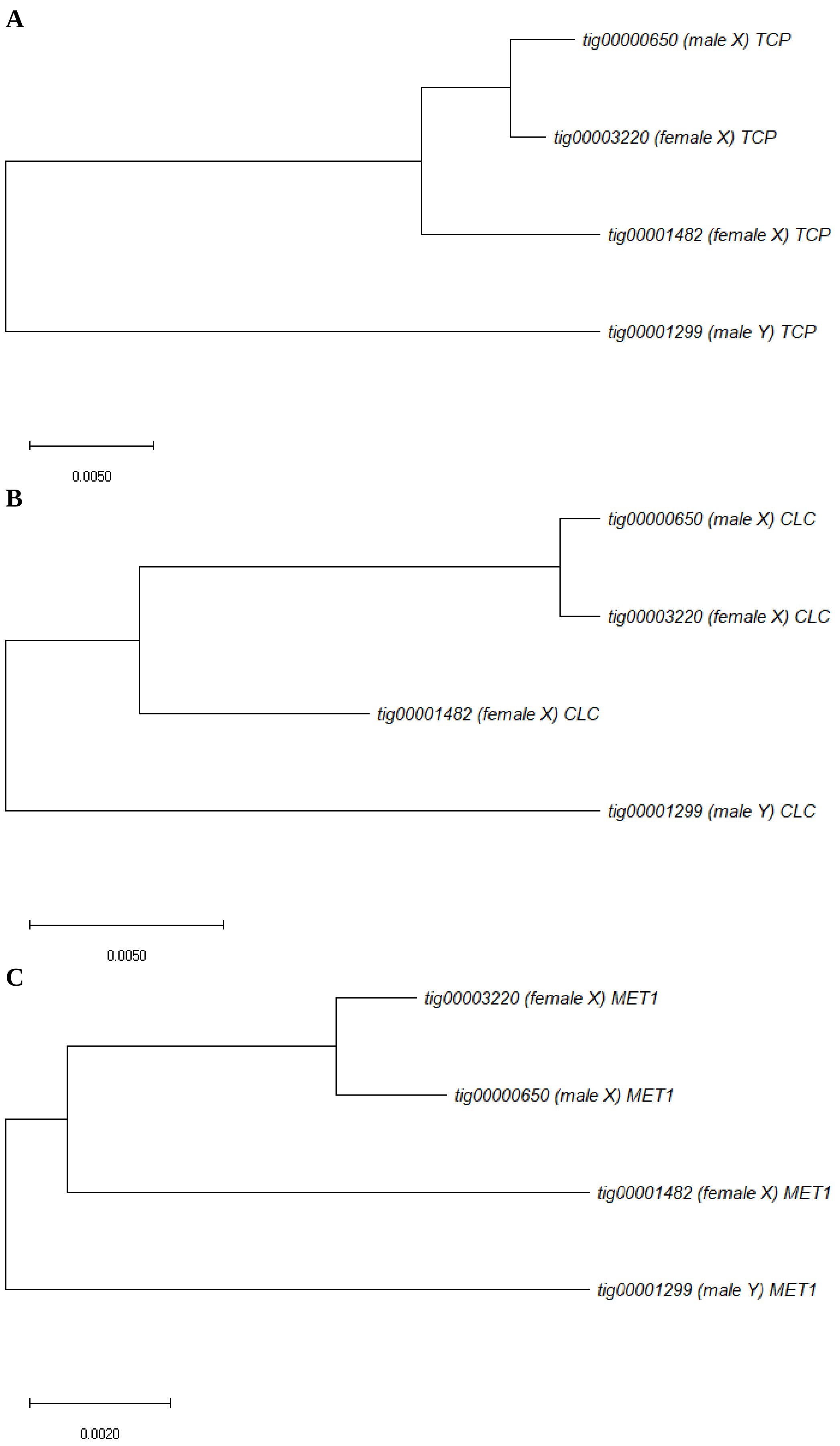

Supplementary Data 4. Clusterization of the *TCP* (**A**), *CLC* (**B**), and *MET1* (**C**) genes of the male (tig00001299 – Y haplotype, tig00000650 – X haplotype) and female (tig00001482 – X haplotype and tig00003220 – X haplotype) *P.* × *sibirica*.
