# Supplementary material for: Genome Assembly and Sex-Determining Region of Male and Female Populus × sibirica
Source: Front Plant Sci. 2021 Sep 8;12:625416. doi: 10.3389/fpls.2021.625416 (PMC8455832; doi:10.3389/fpls.2021.625416)
Supplement: Supplementary Data 5 — Clusterization of the ARR17 gene and five ARR17 partial repeats of males of P. × sibirica, P. trichocarpa, P. deltoides, and P. simonii. In the P. × sibirica genome assembly, two allelic variants of the ARR17 gene were identified [designated ARR17 (c1) and ARR17 (c2)]. ARR17 partial repeat 1 is the closest to the end of the chromosome and the subsequent ones are located further from the end in ascending order, according to the P. trichocarpa “Stettler 14” genome assembly. [file Data_Sheet_5.PDF]

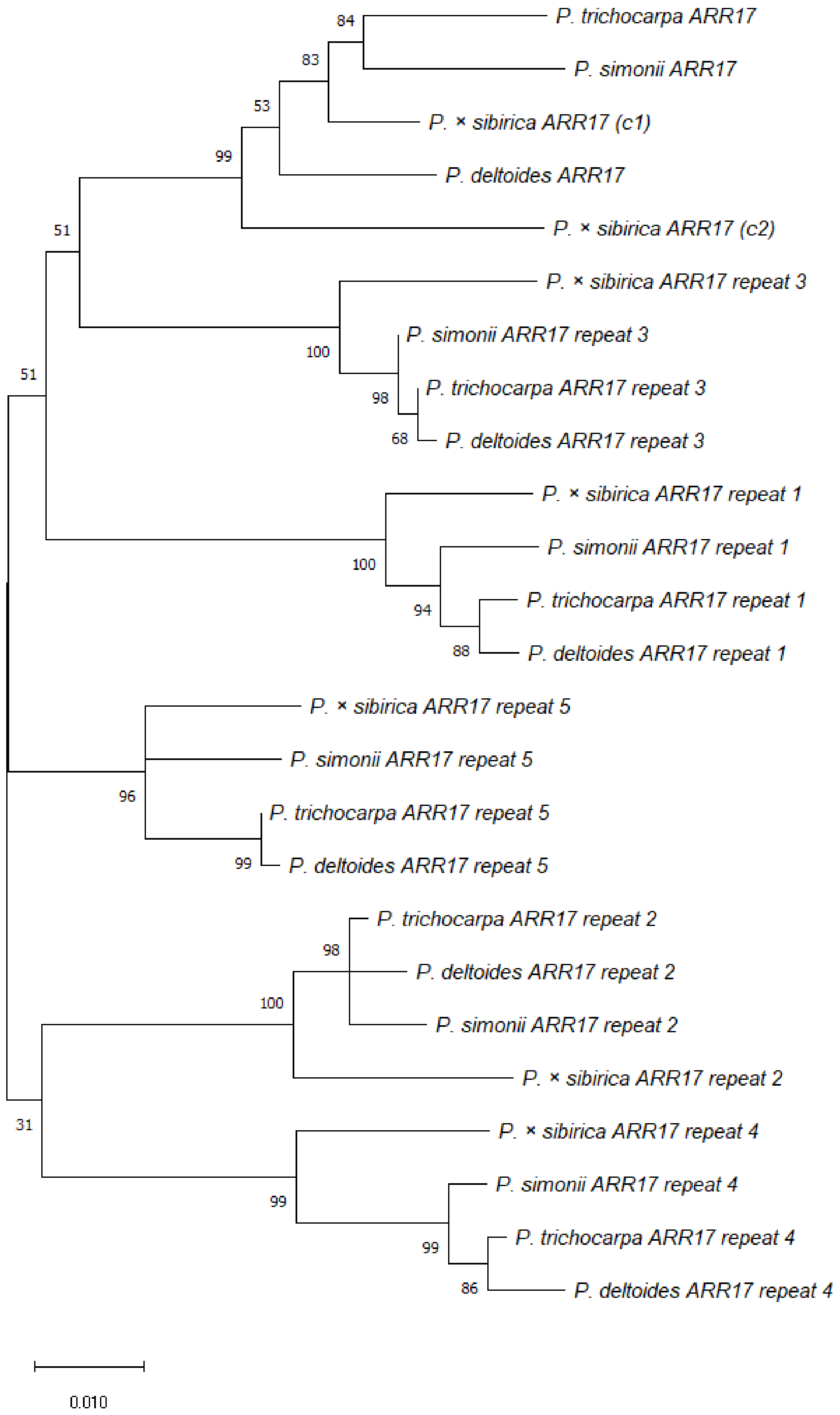

Supplementary Data 5. Clusterization of the *ARR17* gene and five *ARR17* partial repeats of males of *P.* × *sibirica*, *P. trichocarpa*, *P. deltoides*, and *P. simonii*. In the *P.* × *sibirica* genome assembly, two allelic variants of the *ARR17* gene were identified (designated *ARR17* (c1) and *ARR17* (c2)). *ARR17* partial repeat 1 is the closest to the end of the chromosome and the subsequent ones are located further from the end in ascending order, according to the *P. trichocarpa* "Stettler 14" genome assembly.
