# Supplementary material for: Genome Assembly and Sex-Determining Region of Male and Female Populus × sibirica
Source: Front Plant Sci. 2021 Sep 8;12:625416. doi: 10.3389/fpls.2021.625416 (PMC8455832; doi:10.3389/fpls.2021.625416)
Supplement: Supplementary Data 7 — Clusterization of 70 Populus males and the male P. × sibirica based on Illumina WGS data aligned to the male P. trichocarpa “Stettler 14” genome with a further search for polymorphisms in the region of ARR17 partial repeats. [file Data_Sheet_7.PDF]

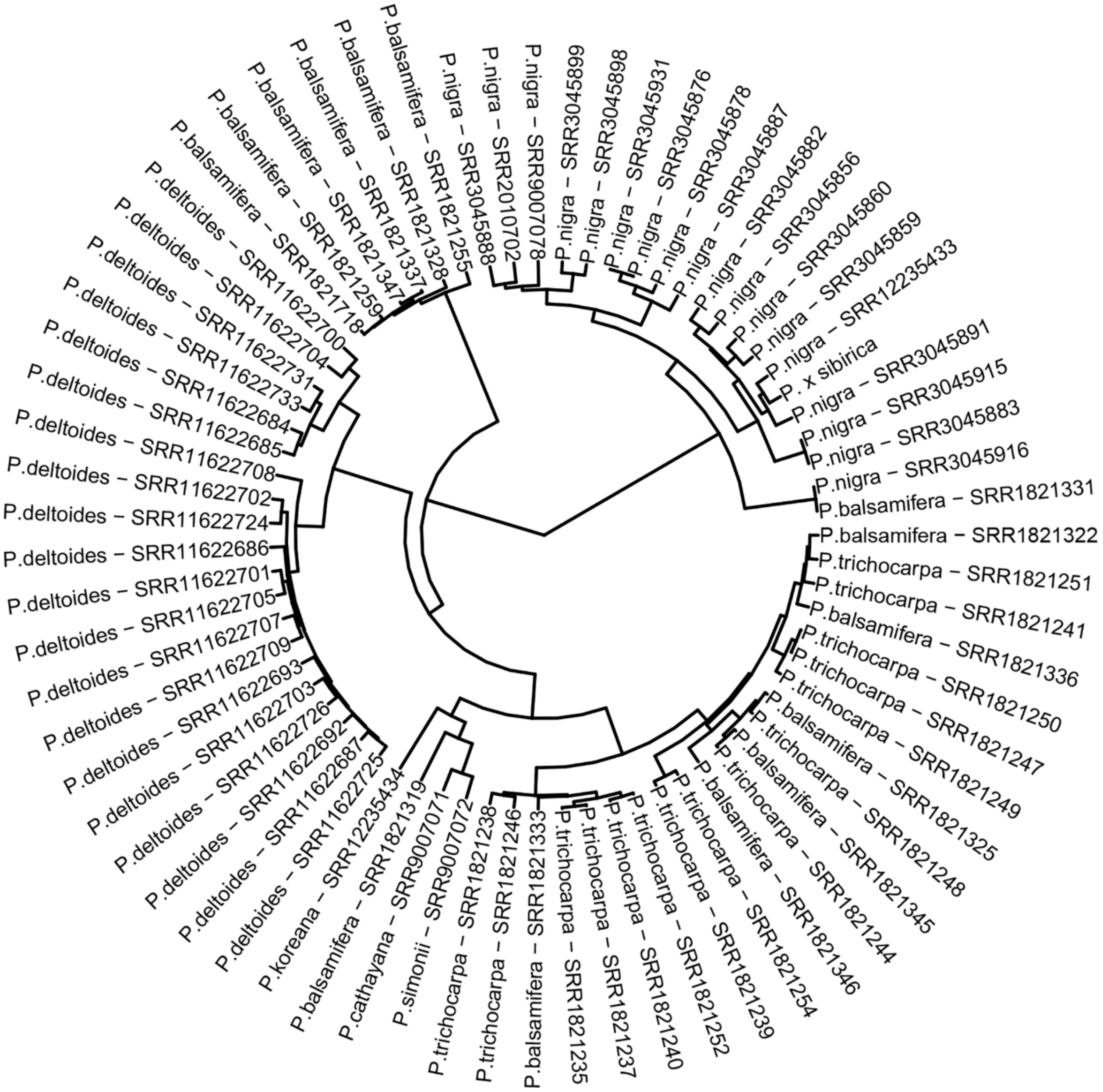


**Supplementary Data 7.** Clusterization of 70 *Populus* males and the male *P.* × *sibirica* based on Illumina WGS data aligned to the male *P. trichocarpa* "Stettler 14" genome with a further search for polymorphisms in the region of *ARR17* partial repeats.
