# Supplementary material for: Genome Assembly and Sex-Determining Region of Male and Female Populus × sibirica
Source: Front Plant Sci. 2021 Sep 8;12:625416. doi: 10.3389/fpls.2021.625416 (PMC8455832; doi:10.3389/fpls.2021.625416)
Supplement: Supplementary Data 9 — Clusterization of 70 Populus males and the male P. × sibirica based on Illumina WGS data aligned to the male P. × sibirica genome assembly with a further search for polymorphisms in the Y SDR haplotype. [file Data_Sheet_9.PDF]

Y SDR haplotype

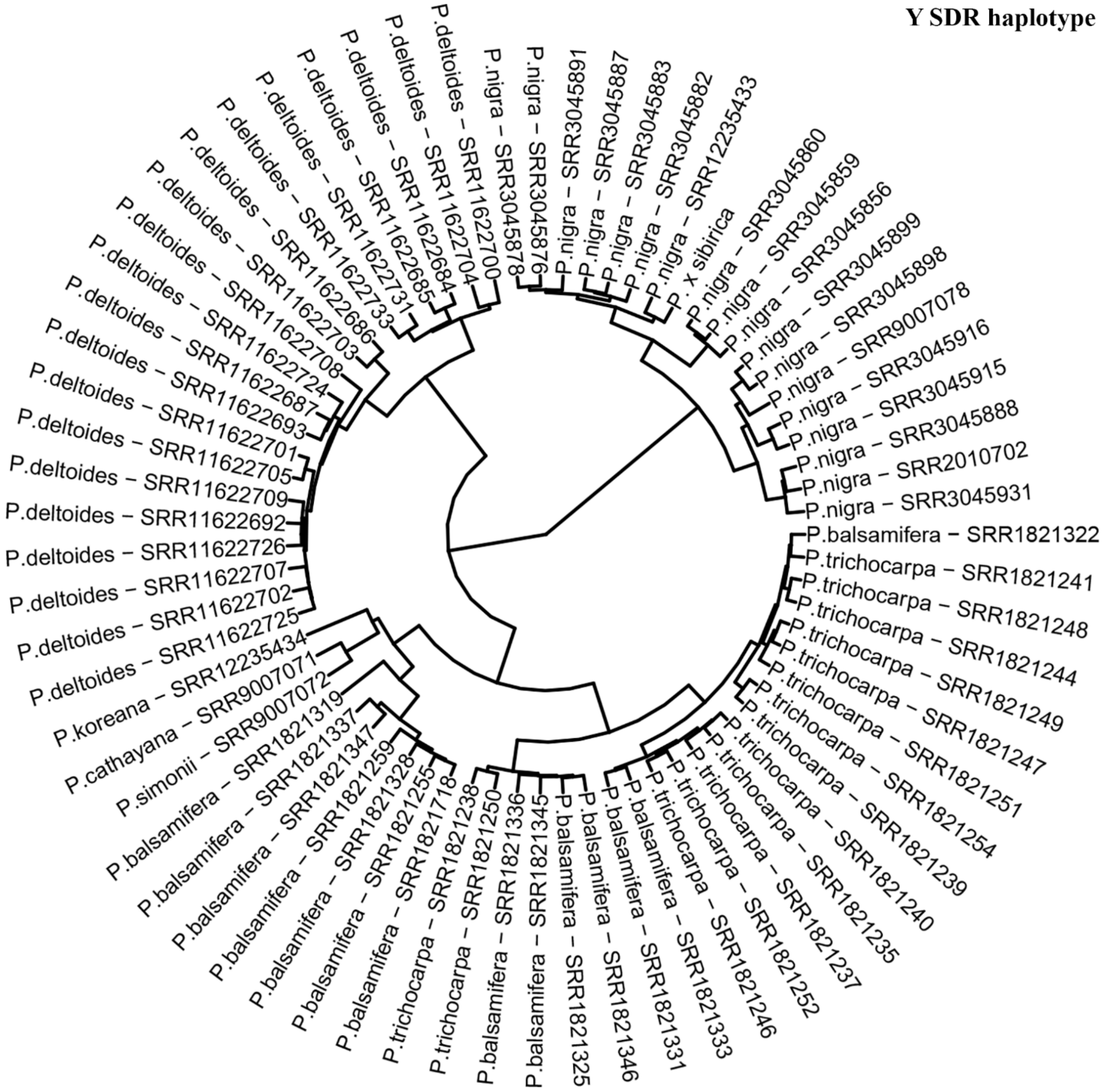

Supplementary Data 9. Clusterization of 70 *Populus* males and the male *P.* × *sibirica* based on Illumina WGS data aligned to the male *P.* × *sibirica* genome assembly with a further search for polymorphisms in the Y SDR haplotype.
